# Fractional 2′-O-methylation in the ribosomal RNA of *Dictyostelium discoideum* supports ribosome heterogeneity in Amoebozoa

**DOI:** 10.1038/s41598-022-05447-w

**Published:** 2022-02-04

**Authors:** Jan Diesend, Ulf Birkedal, Jonas Kjellin, Jingwen Zhang, Kim Philipp Jablonski, Fredrik Söderbom, Henrik Nielsen, Christian Hammann

**Affiliations:** 1 Ribogenetics, Biochemistry Laboratory, Department of Life Sciences and Chemistry, Jacobs University gGmbH, Campus Ring 1, 28759 Bremen, Germany; 2 Department of Cellular and Molecular Medicine, University of Copenhagen, Copenhagen, Denmark; 3 Department of Cell and Molecular Biology, Uppsala University, Box 596, 75124 Uppsala, Sweden; 4 Genomics Group, Nord University, Bodø, Norway; 5 Present Address: Department of Clinical Genetics, Copenhagen University Hospital, Rigshospitalet, Copenhagen, Denmark; 6 Present Address: Health and Medical University GmbH, Villa Carlshagen, Olympischer Weg 1, 14471 Potsdam, Germany

**Keywords:** Biochemistry, Chemical biology

## Abstract

A hallmark of ribosomal RNA (rRNA) are 2′-*O*-methyl groups that are introduced sequence specifically by box C/D small nucleolar RNAs (snoRNAs) in ribonucleoprotein particles. Most data on this chemical modification and its impact on RNA folding and stability are derived from organisms of the Opisthokonta supergroup. Using bioinformatics and RNA-seq data, we identify 30 novel box C/D snoRNAs in *Dictyostelium discoideum*, many of which are differentially expressed during the multicellular development of the amoeba. By applying RiboMeth-seq, we find 49 positions in the 17S and 26S rRNA 2′-O-methylated. Several of these nucleotides are substoichiometrically modified, with one displaying dynamic modification levels during development. Using homology-based models for the *D. discoideum* rRNA secondary structures, we localize many modified nucleotides in the vicinity of the ribosomal A, P and E sites. For most modified positions, a guiding box C/D snoRNA could be identified, allowing to determine idiosyncratic features of the snoRNA/rRNA interactions in the amoeba. Our data from *D. discoideum* represents the first evidence for ribosome heterogeneity in the Amoebozoa supergroup, allowing to suggest that it is a common feature of all eukaryotes.

## Introduction

Early on, the peptidyltransferase reaction of the ribosome was shown to be resistant to protein degradative treatment^1^. This first indication for rRNA as the catalytic entity in protein biosynthesis, rather than proteins, was subsequently confirmed by ground-breaking and highly decorated crystallographic work^2–4^. Maturation of ribosomes is amongst the most complex cellular processes and requires about 200 facilitating proteins, as reviewed recently^5^. Amongst many other processes, the introduction of post-transcriptional, covalent modifications in rRNA is of utmost importance for ribosome biogenesis and function, as summarized in Ref.^6^. The most prominent nucleotide modifications in rRNA are 2′-*O*-ribose methylation (2′-*O*-Me) and pseudouridylation (Ψ) that are introduced site-specifically. These modifications are thought to be important for RNA folding, ribosome stability and translational fidelity^7–9^. In recent years, a specialization of ribosomes in response to environmental changes and/or developmental processes has been suggested, with substoichiometric chemical modifications being implicated as a major source of ribosome heterogeneity^6,10^. As such, examples for fractional rRNA modifications are found in various species, including *Saccharomyces cerevisiae*, where 18 positions are modified in less than 85% of the ribosomal population^11^, and also approximately a third of the 2′-*O*-Me positions in rRNA of *Homo* *sapiens* are found hypomodified^12^. Recently, altered 2′-*O*-Me levels were also discovered during the development of *Danio rerio*^13^. Functionally, ribosome heterogeneity has been proposed to constitute a fine-tuning mechanism for translational activity of an unknown subset of mRNAs^14,15^.

**Figure 1:**
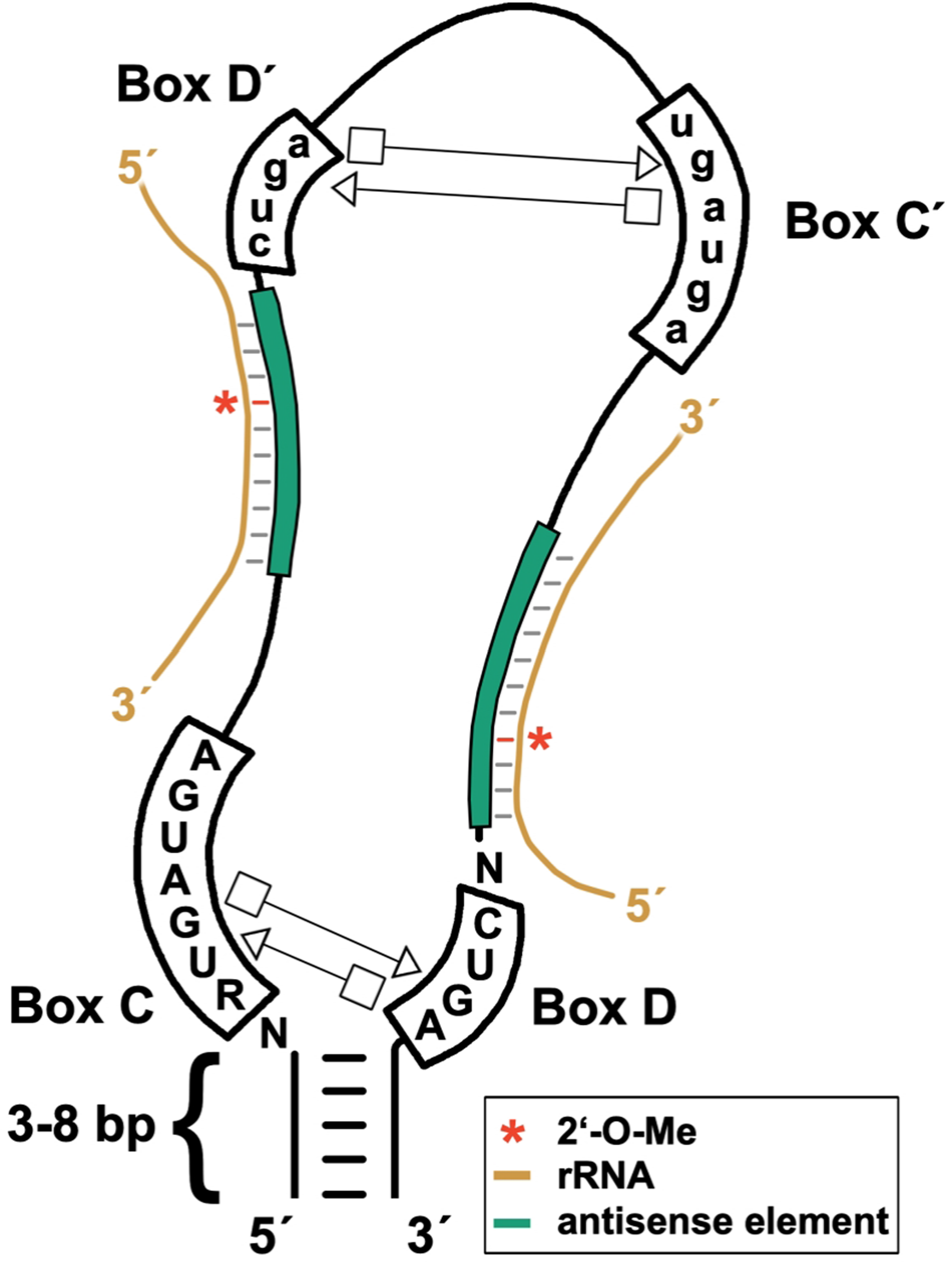
Features of box C/D snoRNAs. Conserved residues of boxes C and D are shown. They interact to form a functionally important k-turn by means of *trans* Hoogsteen/sugar-edge A•G base pairs, shown in conventional Leontis–Westhof symbols^75^. The guiding sequences (“antisense element”; green) is upstream of the D box with methylation occurring in rRNA at the position pairing to the 5th nucleotide upstream of the D box (indicated with a red asterisk). Base pairing with rRNA (beige) is schematically shown. Boxes C′ and D′ are usually less well conserved (indicated by small lettering). The separate antisense sequence upstream of box D′, allows guidance to a further methylation site.

Ribose methylations and pseudouridylations in eukaryotes are introduced in rRNA site-specifically by small nucleolar ribonucleoprotein particles (snoRNPs), as summarized recently^6,10,16^. They come in two flavors: H/ACA snoRNPs catalyze the conversion of uridine to Ψ, while box C/D snoRNPs introduce methyl groups at the 2′-hydroxyl of ribose residues^17,18^. For each class of snoRNPs, a conserved and distinct set of four proteins form the catalytic complex, of which dyskerin in the H/ACA snoRNPs isomerizes uridine^19^, while fibrillarin in box C/D snoRNPs acts as the methyltransferase on 2′-hydroxyl groups^20^. The rRNA target positions are defined by the individual snoRNA components of the RNPs. For both classes, specific base pairing patterns define the nucleotide to be modified. In the following, we briefly summarize the interaction of box C/D snoRNAs with rRNA and refer for H/ACA snoRNAs to two recent excellent reviews^6,10^. Box C/D snoRNAs possess conserved box C (5′-RUGAUGA-3′) and box D (5′-CUGA-3′) motifs that are essential for their structure, function and biogenesis (Fig. 1), as well as less conserved box C′ and box D′ motifs^21–23^. Nucleotides of the box C and box D motifs interact with each other, forming a kink-turn, and similar, but weaker interactions may also occur between nucleotides of the box C′ and box D′ motifs. The intramolecular base pairs of box C and box D motifs are essential for snoRNA processing and the snoRNP structure. Immediately upstream of the D and/or D′ box are antisense elements that base pair with the rRNA target and thereby direct fibrillarin to its site of action. In an archaeal model for box C/D sRNPs, the substrate-binding channel of the complex accommodates 10 base pairs of the snoRNA/rRNA duplex^24^. Methylation occurs at the 2′-hydroxyl of the nucleotide base paired to the 5th nucleotide upstream of the D or D′ box in an *S*-adenosylmethionine (SAM)-dependent reaction^20,25,26^. Since both, the D and D′ box can potentially guide 2′-*O*-Me with their antisense elements, these snoRNAs can guide in principle two sites in rRNA. Furthermore, a reorientation of the box C/D snoRNA inside the snoRNP complex might lead to alternative base pairing, increasing the target number of a given snoRNA even further^27,28^.

While a substantial amount of data on rRNA modifications by snoRNPs is available from organisms of the evolutionary supergroups of Opisthokonta and Archaeplastida^11–13,29,30^, information for the Amoebozoa supergroup remains scarce. *Dictyostelium discoideum* is a well-established model organism and arguably the best studied organism of the Amoebozoa^31^. A wide spectrum of experimental tools has been established for the amoeba^32^, and these are frequently used to study mechanisms governing cell motility, autophagy, social evolution (reviewed in Ref.^33^), mobile genetic elements^34^, and their domestication by the RNA interference (RNAi) machinery^35–37^. *D.* *discoideum* single cells usually propagate by mitotic division; upon starvation, however, a complex developmental process is initiated, in which about 100,000 cells aggregate to form a multicellular mobile slug after 16 h, resulting in a fruiting body within 24 h^38^. This allows to study fundamental developmental processes in the amoeba.

In most metazoans, the genes for the rRNAs are organized in rDNA clusters, an arrangement that is thought to facilitate efficient rRNA transcription. Such rDNA clusters exist also in *D. discoideum*, however, they are not encoded in chromosomes but localized on extrachromosomal elements^39,40^. Each nucleus contains about 100 copies of these elements of 88 kb, that each feature two rRNA transcription units organized as palindromes^41^. A first model for the processing of rRNAs from the primary 37S transcript in the amoeba has been proposed, and sequences of the mature rRNAs in *D. discoideum* were determined experimentally^42^. Earlier work has identified several box C/D snoRNAs in *D. discoideum*, and verified a function in rRNA 2′-O-methylation^43^. The study employed a shotgun cloning approach to identify novel non-coding RNAs (ncRNAs) in *D. discoideum*. This work led also to the discovery of the functionally important Class I RNAs, which recently were shown to be involved in the evolution of multicellularity in Dictyostelia^44^. Next to these, sequencing of cloned fragments yielded 17 box C/D and one box H/ACA snoRNA(s) in *D. discoideum*, besides other ncRNAs.

Owing to these observations, we set out here to elucidate the global 2′-*O*-Me pattern(s) in the amoeba. Employing RiboMeth-seq (RMS)^11^, we created a comprehensive map of the 2′-*O*-Me sites in *Dictyostelium*’s 17S and 26S rRNAs. We thereby positioned methylated residues in functional important parts of the rRNAs, for which we have determined sequence homology-based models of their secondary structures. Further, we also have identified bioinformatically and validated experimentally additional box C/D snoRNAs with which we can at large explain methylated rRNA positions in the amoeba.

## Methods

### Cell culture and growth conditions of *D. discoideum*

The *D. discoideum* strains AX2^45^ and *∆drnB*^46^ were cultivated in HL5 medium containing 50 μg/mL ampicillin, 250 ng/mL amphotericin, 500 U/mL penicillin/streptomycin at 22 °C in shaking suspension.

### Filter development of *D. discoideum*

Filter development was performed using 5 × 10^8^ of axenically grown *D. discoideum* cells pelleted for 5 min at 500×*g* and washed three times with Sørensen buffer [2 mM Na_2_HPO_4_, 15 mM KH_2_PO_4_, (pH 6.7)]. The pellet was resuspended in Sørensen buffer and transferred in a 6-cm dish containing two layers of Whatman^®^ paper topped off with a nitrocellulose membrane. After 16 h, the slugs were harvested by washing the nitrocellulose membrane with Sørensen buffer and spun down by centrifugation at 500×*g* for 5 min. RNA was isolated from the resulting pellet.

### Resources for RNA-seq datasets

RNA-seq datasets of AX2 and *∆drnB* in axenic growth and slug stage of development were acquired from the sequence read archive (https://www.ncbi.nlm.nih.gov/sra) and used for RNA-seq validation of box C/D snoRNA candidates and expression analysis. Accession numbers of the utilized data sets can be found in Supplementary Table S1. Sample preparation and sequencing was described in Liao et al.^47^.

### In silico identification and validation of box C/D snoRNA candidates

The genomic sequences were retrieved from Dictybase (www.dictybase.org) and the sequences of the 17S and 26S rRNA^42^ were retrieved from GenBank (www.ncbi.nlm.nih.gov/genbank/; Accession numbers are listed in Supplementary Table S3). The identification of box C/D snoRNA candidates in *D. discoideum* was performed using snoScan v. 0.9.1^48^ with threshold settings (− C 0 − D 0 − X 0) disabled. Candidates with a combined box C and box D score higher than 9 and a box C-D distance between 50 and 100 nt were selected for RNA-seq validation. Sequencing reads from the axenic AX2 dataset were aligned to the genomic coordinates ± 150 bp using bowtie v. 1.2.3^49^ allowing for one mismatch. Box C/D snoRNAs were considered validated, if reads specifically matched the predicted loci and read coverage calculated with BEDTools coverage v. 2.29.2^50^ indicated a distinct 5′ end, yielding an expression score of 15. Box C/D snoRNA candidates lacking expression or a distinct 5′ end received a penalty of − 15. All scores were combined into a classifier score containing C/D box scores, terminal stem score, Box C–D distance score, and the expression score (Supplementary Fig. S1). If a total classifier score of 29 or higher was achieved, the candidate was considered to be an expressed *bona fide* box C/D snoRNA and kept for further analyses and assignment to the predicted ribosomal 2′-*O*-Me pattern.

### RNA-seq analysis of box C/D snoRNAs in development

Reads were aligned using bowtie v. 1.2.3^49^ allowing for one mismatch and counted with featureCounts v. 2.0.0^51^. Between-sample normalization was done by DEseq2 v. 1.29.6^52^. P-values were adjusted using the false discovery rate (FDR) method. Principal component analysis was performed on DESeq2-normalized reads using R-stats v. 4.0.0 and visualized with R-ggplot2 v. 3.3.2. The heatmap of log_2_ fold-change of box C/D snoRNAs was generated using ComplexHeatmap v. 2.5.3^53^.

### Radiolabeling of DNA oligonucleotides

DNA oligonucleotides were purchased from Merck and are listed in Supplementary Table S2. For primer extension and northern blot analysis, 10 pmol oligonucleotide was 5′-end-labeled by incubation with 10 U T4 polynucleotide kinase (Fermentas) for 30 min at 37 °C in 50 mM Tris–HCl (pH 7.6), 10 mM MgCl_2_, 5 mM DTT, 100 µM spermidine, and 0.37 MBq [γ-^32^P]-ATP. The reaction was stopped at 80 °C for 5 min, the radiolabeled oligonucleotides were phenol/chloroform-extracted and purified using a Sephadex G50 (GE Healthcare) column.

### RNA extraction

RNA was isolated from 2 × 10^7^ axenically grown *D. discoideum* cells washed with pre-cooled Sørensen buffer [2 mM Na_2_HPO_4_, 15 mM KH_2_PO_4_, (pH 6.7)]. Cells were pelleted and resuspended in TRIzol reagent (Invitrogen) containing 10 mM EDTA (pH 8.0). RNA was extracted according to the manufacturer’s instructions. RNA concentration was determined spectrophotometrically.

### Primer extension

For primer extension, a box C/D snoRNA-specific 5′-radiolabeled oligonucleotide was annealed to 4 µg RNA at 65 °C for 5 min and cooled for at least 1 min on ice. Upon annealing, 1× SuperScript IV buffer (ThermoFisher Scientific, Inc.), 1 mM dNTP mix, 5 µM DTT, 40 U RiboLock RNase Inhibitor (ThermoFisher Scientific, Inc.) and 50 U SuperScript IV Reverse Transcriptase (ThermoFisher Scientific, Inc.) were added. The reaction was incubated at 55 °C for 30 min and stopped at 85 °C for 5 min. Products were phenol/chloroform-extracted, recovered by ethanol precipitation and separated on a polyacrylamide gel (12% PAA, 20 mM MOPS, pH 7.0, 7 M Urea) for 3 h at 25 mA.

### Northern Blot analysis

For the detection of snoRNAs, 20 μg of total RNA was separated by gel electrophoresis on a 12% polyacrylamide gel (20 mm MOPS, pH 7.0, 7 M urea). The RNA was transferred to a nylon membrane (Amersham Biosciences Hybond^TM^-NX) by electroblotting for 30 min at 20 V. Blotted RNA was crosslinked by 0.5 J/cm^2^ UV illumination. Blots were probed overnight with 5′-radiolabelled DNA oligonucleotides in Church buffer (1 mM EDTA, 7% (w/v) SDS, 1% (w/v) BSA in 0.5 M P_i_ buffer, pH 7.2). Probed Blots were washed two times for 20 min with each 2×, 1×, and 0.5× SSC buffer (20× SSC: 3 M NaCl, 0.3 M trisodium citrate, pH 7.0). Hybridization with an oligonucleotide complementary to tRNA_UUC_ was used as a loading control.

### RiboMeth-seq

The RiboMeth-seq analysis was performed in triplicates with barcoded adapters according to previously described protocols^11,54^. In brief, 10 µg RNA from each sample was degraded by alkaline for 6 min at 90 °C and the 20–40 nt fraction was excised and purified from a 10% urea polyacrylamide gel. A modified *Arabidopsis* tRNA ligase was used to ligate adaptors to the library fragments, and sequencing was carried out on the Ion Proton sequencing platform. The reads were mapped to rRNAs (GenBank: FR733593.1, FR733594.1, FR733597.1, FR733595.1) using Bowtie2^55^ and scored for read-end counts. RMS scores representing “fraction methylated” were calculated as described previously (“score C”) in Ref.^11^ and barcode correction was applied when necessary^56^. The commercial RNA oligonucleotides used as 3′adaptors were found to be slightly heterogeneous in length, which can cause a fractional shift in the 3′-read-end count, if the 3′-library fragment nucleotide is identical to the expected 5′-end of the oligonucleotide. As the experiments were made in triplicate with barcodes carrying different 5′-ends, such errors were easily detected, and a manual correction was made at a few sites to counter the effect by excluding the 3′-read-end counts from the analysis.

### Prediction of rRNA secondary structure

To locate the predicted 2′-*O*-Me sites in the mature rRNA, we predicted the secondary structure by comparative analysis with the LSU and SSU rRNAs of *A. thaliana*, *C. elegans*, *H. sapiens*, and *D. melanogaster*. For that purpose, we retrieved the corresponding SSU and LSU rRNA sequences for these organisms from GenBank (Supplementary Table S3). We aligned the sequences to the 17S and 26S rRNA of *D. discoideum* using MUSCLE^57^ in the ClustalW output format and inferred the secondary structure by homology manually. The resulting secondary structure diagrams were drawn using RNAviz v. 2.0.3^58^. Due to the high conservation of the ribosomal core elements and experimental evidence of the tRNA site locations in other species, the nucleotides predicted in the A, P, and E sites of *D. discoideum* were inferred by sequence homology.

### Mapping of predicted snoRNA candidates to the rRNA 2′-*O*-Me pattern

Mapping of box C/D snoRNAs to the predicted 2′-*O*-Me sites was performed using RNAhybrid^59^. 10 nt upstream and downstream of the 2′-*O*-Me sites were used as target sites against the full-length sequences of the box C/D snoRNAs. Selection of the likely correct duplex was achieved using the following criteria: (I) 2′-*O*-Me site is located at the 5th base paired nucleotide upstream of a D or D′ box and (II) a box C/D snoRNA/rRNA duplex length of minimum 7 bp with (III) a maximum of 1 mismatch. Conservation of box C and box D motifs was visualized using WebLogo v. 3.7^60^. Calculation of the predicted duplex’ minimum free energy (MFE) in kcal/mol was performed using RNAduplex v. 2.4.15^61^. Box C/D snoRNAs that were not mapped to any predicted 2′-*O*-Me sites but were validated by RNA-seq, were classified as orphans.

## Results

### Identification and validation of 30 novel box C/D snoRNAs in the genome of *D. discoideum*

The number of 17 box C/D snoRNAs (Fig. 1) identified in *D. discoideum* prior to this study is relatively small for normally-sized rRNA sequences^42^ compared to orthologous RNAs found in other species^62^. Therefore, we set out here to search for additional box C/D snoRNAs in the amoeba. To this end, we employed an *in silico*-approach for the identification of novel box C/D snoRNAs by using the probabilistic model-dependent search tool snoScan^48^, which we combined with RNA-seq analyses. The sizes of previously described box C/D snoRNAs of *D. discoideum* range between 66 and 113 nt, with box C-D distances between 50 and 97 nt^43^. We searched accordingly first with snoScan in the genome of *D. discoideum* (available at www.dictybase.org) for sequences containing box C and box D motifs with a box C-D distance between 50 to 100 nt. Since inverted repeats at the 5′ and 3′ ends were not observed before^43^, we did not pre-require the presence of a terminal stem structure for a classification as a *bona fide* box C/D snoRNA. Using these settings, we identified 577 box C/D snoRNA candidates in the genome of *D. discoideum* (data not shown), including the set described before^43^. To refine our search, we next addressed the expression of these candidates in publicly available RNA-seq data of the axenic AX2 wild type strain, deposited in duplicate^47^ at the sequence read archive (https://www.ncbi.nlm.nih.gov/sra). Specifically, we mapped reads to the genomic loci of the candidates and selected only those sequences that exceeded a read count of 100 and were not part of a longer transcript, as indicated by a distinct 5′ end. Both, the lack of specific RNA-seq reads or of a distinct 5′ end, were penalized (‘expression score’, Supplementary Fig. S1). Sequences scoring 29 or higher in the classifier score (Supplementary Table S4) were classified as *bona fide* box C/D snoRNAs. This routine allowed us to identify 47 box C/D snoRNAs in *D. discoideum*, of which 30 are novel^43^. For the amoeba, box C/D snoRNA gene clusters have been described^43^ and primary transcripts of such clusters are often processed by an RNase III before exonucleolytic processing can occur^63–65^. We therefore included the knock-out strain of the nucleolar RNase III DrnB^46,66,67^ in the following analyses. Initially, we carried out primer extension experiments on RNA isolated from axenically grown or developed AX2 and *∆drnB* cells. This resulted for the majority of the snoRNAs in a single signal at the predicted size (Supplementary Fig. S2), indicating that they have homogeneous 5′-ends. Their genomic locations are listed in Supplementary Table S5, allowing to characterize next the properties of box C/D snoRNA genes in *D. discoideum*.

### The box C/D snoRNA genes in *D. discoideum*

Usually, box C/D snoRNAs are encoded in intergenic regions or as part of introns in protein-coding genes, and in either set-up, they can be generated as mono- or poly-cistronic transcriptional units^62^. Aspegren et al.^43^ predicted four bi-cistronic transcriptional units of snoRNAs in *D. discoideum* and confirmed expression for several of them using RT-PCR. An analysis of the genomic location of the genes for our set of 47 box C/D snoRNAs revealed five additional clusters containing two box C/D snoRNAs and two clusters comprised of three box C/D snoRNAs (Supplementary Fig. S3). The genes for these box C/D snoRNAs appear equally spaced in the clusters. All box C/D snoRNA genes, in clusters or not, were found in intergenic regions, except CD38, which is encoded in an intron (Supplementary Table S5). The box C/D snoRNAs with a predicted target (see below) are encoded on all chromosomes without a noticeable pattern, but we observed that the majority of box C/D snoRNAs without a target are encoded on chromosome 4. The biological significance of this, if any, remains to be elucidated, and we cannot exclude that it is a random localization. Next, we set out to investigate the 2′-*O*-Me patterns in *D. discoideum*’s rRNAs, that would be guided by the encoded box C/D snoRNAs.

### *Dictyostelium discoideum* 17S and 26S rRNAs have 49 high-confidence 2′-*O*-Me sites

To address 2′-*O*-Me in the 17S and 26S rRNA of *D. discoideum*, we employed RMS, a method introduced on yeast rRNA^11^, and subsequently used in several other organisms^12,13,30^. In brief, RMS is a next-gen sequencing-based method that relies on the cleavage-resistance of 2′-O-methylated nucleotides under alkaline conditions, resulting in an underrepresentation of read ends in fragmented RNA. The results are expressed as RMS scores, which represent the fraction of modified molecules at a given position. The method yields methylation stoichiometry comparable to RP-HPLC^68^. We generally considered sites with an RMS score > 0.75 as high-confidence 2′-*O*-Me sites.

**Figure 2:**
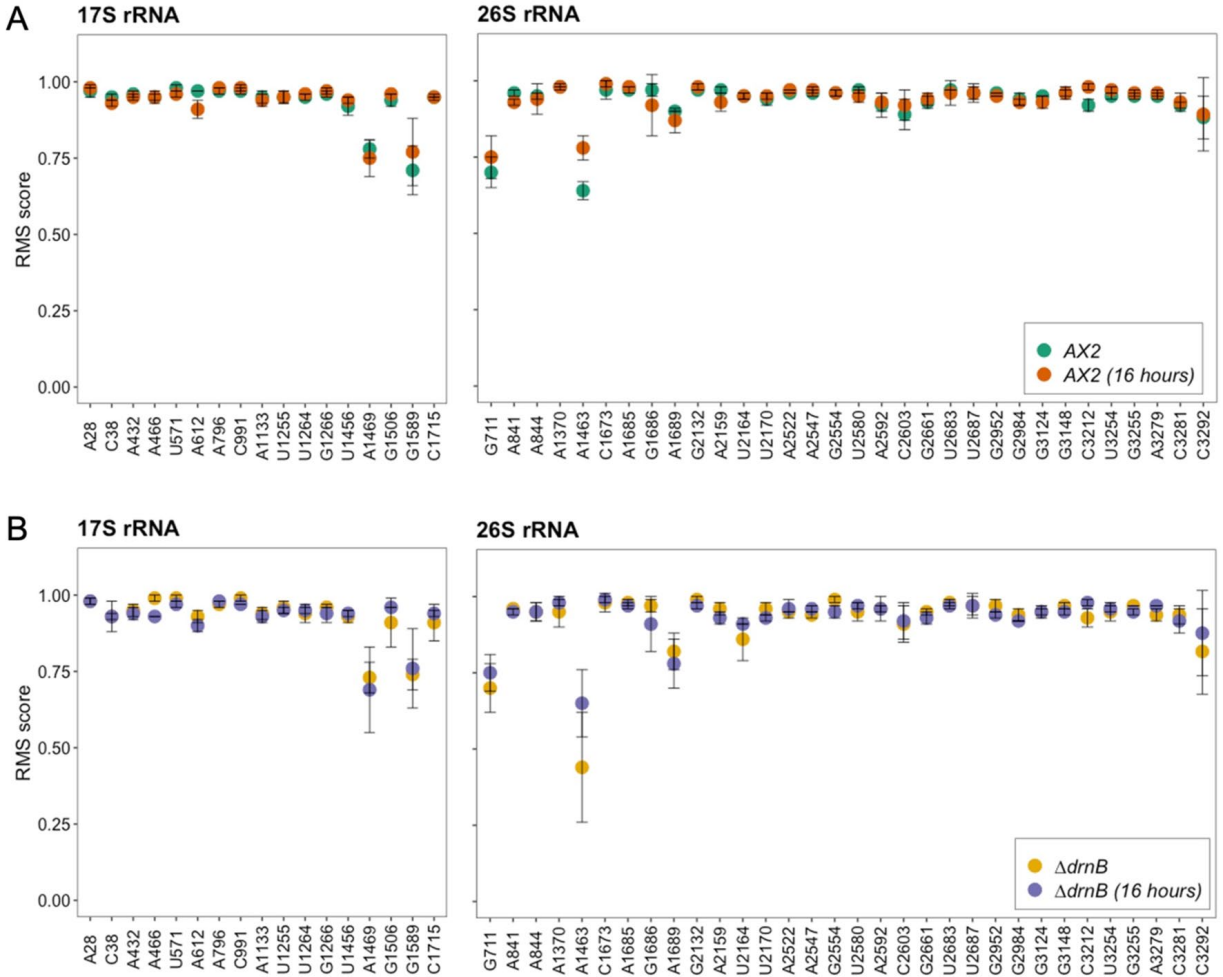
RiboMeth-seq analysis of the 17S and 26S in *D. discoideum*. RMS scores at 2′-*O*-Me sites on the 17S and 26S rRNA in axenic growth and development of AX2 (**A**) and *∆drnB* (**B**) cells (n = 3).

To investigate the global 2′-*O*-Me landscape in wild type *Dictyostelium*, we initially determined the RMS scores of rRNA isolated from axenic AX2 cells. During these experiments, we realized that one nucleotide (C784) was missing in the 17S reference sequence^42^, and its presence was independently confirmed by sequencing of a PCR product on total DNA. Using the criteria outlined above, we determined in total 17 and 32 positions with a 2′-*O*-Me moiety on the 17S rRNA and the 26S rRNA, respectively (Fig. 2A). Of these high-confidence sites, the majority appeared to be fully methylated. In axenically-grown AX2 cells, we identified 2 hypomethylated positions each in the 17S and 26S rRNAs. This indicates, to our knowledge for the first time, heterogeneity of the ribosome population in *D. discoideum*. Heterogeneity in rRNA modifications had been, however, reported previously for mouse, human, thale cress, and zebrafish^12,13,29,30,69,70^. In these studies, differences in the ribosome 2′-*O*-Me patterns between cultured cells and differentiated tissues, or during development have been described. Since *D. discoideum* undergoes development upon starvation, we set out next to elucidate any changes of the 2′-*O*-Me pattern in rRNAs of the slug stage of development in the AX2 wild type. The fractionally methylated positions in axenically-grown wild type cells were also substoichiometrically methylated during development, while the RMS score of most 2′-*O*-Me sites remained unchanged (Fig. 2A).

If the nucleolar RNase III DrnB^46,66,67^ is involved in box C/D snoRNA maturation, a knockout strain of its gene might display altered RMS scores, which we investigated next. At large, the 2′-*O*-Me pattern of the AX2 strain, however, was also observed for axenic growth and development of the *∆drnB* strain (Fig. 2B and Supplementary Fig. S4). Only one position, 26S-Am1463, exhibited a noticeable difference between the axenically-grown AX2 and *∆drnB* strains (Supplementary Fig. S4C). This indicates that any effect that DrnB might have on the processing of box C/D snoRNA precursors does not manifest substantially in altered 2′-*O*-Me patterns. Similarly, that position 26S-Am1463 displayed different RMS scores between axenic growth and the slug stage in both, the AX2 and *∆drnB* strains (Supplementary Fig. S4). The four 2′-O-methylated residues that we found either fractionally modified or changed in development had no orthologous modified sites in *S. cerevisiae*, *H. sapiens*, and *A. thaliana* (Table 1, and see below).

**Table 1:** Sites of 2′-O-methylation in rRNA with guiding box C/D snoRNAs in *D. discoideum* and further species.

*D. discoideum*^a^		*S. cerevisiae*^b^		*H. sapiens*^c^		*A. thaliana*^d^	
Position	Guided by	Position	Guided by	Position	Guided by	Position	Guided by
**SSU**^e^							
Am28	CD18	Am28	snR74	Am27	U27	Am28	AtU27
Cm38	CD35	–		–		–	
Am432	CD8	Am436	snR87	Am484	U16	Am438	AtU16
Am466	CD8	–		–		–	
Um571	CD21	Um578	snR77	Um627	HBII–135	Um580	AtsnoR77Y
Am612	CD25	Am619	snR47	Am668	U36A/B	Am621	AtU36
Am796	CD19	–		–		–	
Cm991	CD7	–		–		–	
Am1133	CD10	–		–		–	
Um1255	CD20	–		–		–	
Um1264	CD29	Um1269	snR55	Um1326	U33	Um1270	AtsnoR34
Gm1266	CD37	Gm1271	snR40	Gm1328	U232A	Gm1272	AtsnoR21
Um1456	CD1	–		Um1442	U61	Um1281	AtU61
Am1469		–		–		–	
Gm1506	CD1	Gm1428	snR56	Gm1490	U25	Gm1431	AtsnoR19
Gm1589	CD16 (+ 6)^f^	–		–		–	
Cm1715	CD28	Cm1639	snR70	Cm1703	U43	Cm1641	AtU43
**LSU**^e^							
Gm711	CD7	–		–		–	
Am841	CD12	–		–		–	
Am844	CD24	Am649	U18	Am1313	U18A/B/C	Am647	AtU18
Am1370	CD9/13	Am1133	snR61	Am1858	U38A/B	Am1140	AtU38
Am1463	CD9/13	–		–		–	
Cm1673	CD19	Cm1437	Um24	Cm2338	U24	Cm1439	AtU24
Am1685	CD19	Am1449	Um24	Am2350	U76	Am1451	AtU24
Gm1686	CD16	Gm1450	Um24	Gm2351	U24	Gm1452	
Am1689	CD33	–		–		–	
Gm2132	CD1	–		–		–	
Am2159	CD27	–		–		–	
Um2164	CD31	–		–		–	
Um2170	CD17/32	Um1888	snR62	Um2824	U34	Um1882	AtU34
Am2522	CD4	Am2256	snR63	Am3739	U46	–	
Am2547		Am2281	snR13	Am3764	U15A/B	Am2271	AtU15
Gm2554	CD14	Gm2288	snR75	–		Gm2278	AtU15
Um2580	CD12/34	–		–		–	
Am2592	CD34	–		–		–	
Cm2603	CD26	Cm2337	snR64	Cm3820	U74	–	
Gm2661	CD2/3	–		–		–	
Um2683	CD30	Um2417	snR66	–		–	
Um2687	CD22	Um2421	snR78	Um3904	U52	Um2411	AtsnoR37
Gm2952	CD13	Gm2619	snR67	Gm4166	U31	Gm2610	AtsnoR35
Gm2984	CD5	–		–		–	
Gm3124	CD36	–		–		–	
Gm3148	CD6	Gm2815	snR38	Gm4362	snR38A/B/C	Gm2805	AtsnoR38Y
Cm3212	CD38	–		–		–	
Um3254	CD25	Um2921	snR52	Um4468		–	
Gm3255		Gm2922	Spb1	Gm4469		–	
Am3279	CD11	Am2946	snR71	Am4493	U29	Am2936	AtU29
Cm3281	CD15	Cm2948	snR69	–		–	
Cm3292	CD23	Cm2959	snR73	Cm4506	U35A/B	Cm2949	AtU35

^a^This study.

^b^https://people.biochem.umass.edu/fournierlab/snornadb/mastertable.php.

^c^https://www-snorna.biotoul.fr/human_yeast/.

^d^https://ics.hutton.ac.uk/cgi-bin/plant_snorna/home.

^e^*SSU* small subunit, *LSU* large subunit.

^f^(+ 6) denotes a deviation of the + 5 consensus methylation target site.

### Secondary structure models for the small and large ribosomal subunits in Amoebae

**Figure 3:**
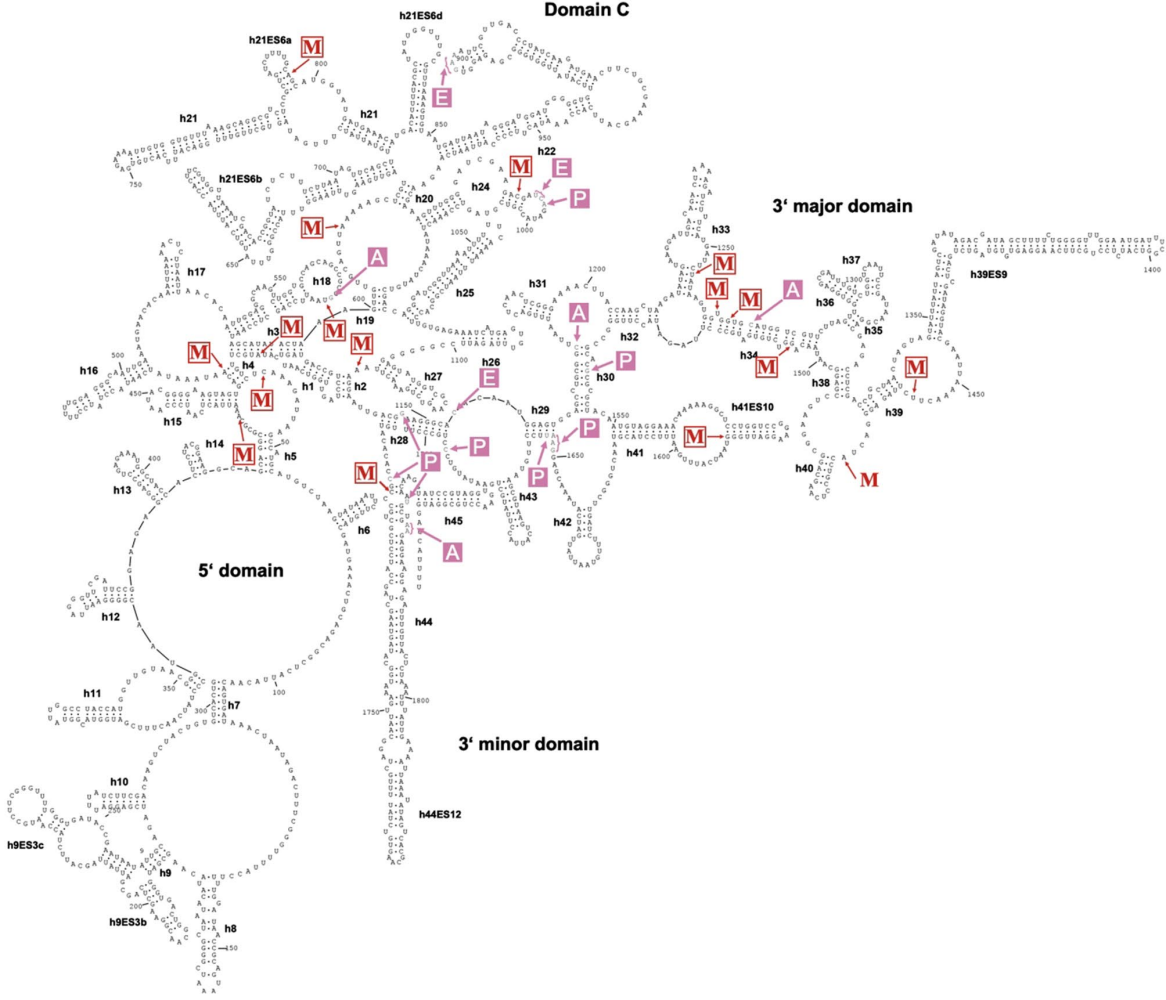
Secondary structure of the 17S rRNA of *D. discoideum* with 2′-*O*-Me sites. The secondary structure of the 17S rRNA was inferred by homology and drawn using RNAviz (v. 2.0.3). The 2′-*O*-methylated nucleotides as identified by RiboMeth-seq are marked with an arrow and ‘M’ (red). Nucleotides located in the A, P, and E sites of the ribosome are indicated in pink. Helices (hx) are named to convention and expansion segments (ESx) are labeled with x: natural number.

**Figure 4:**
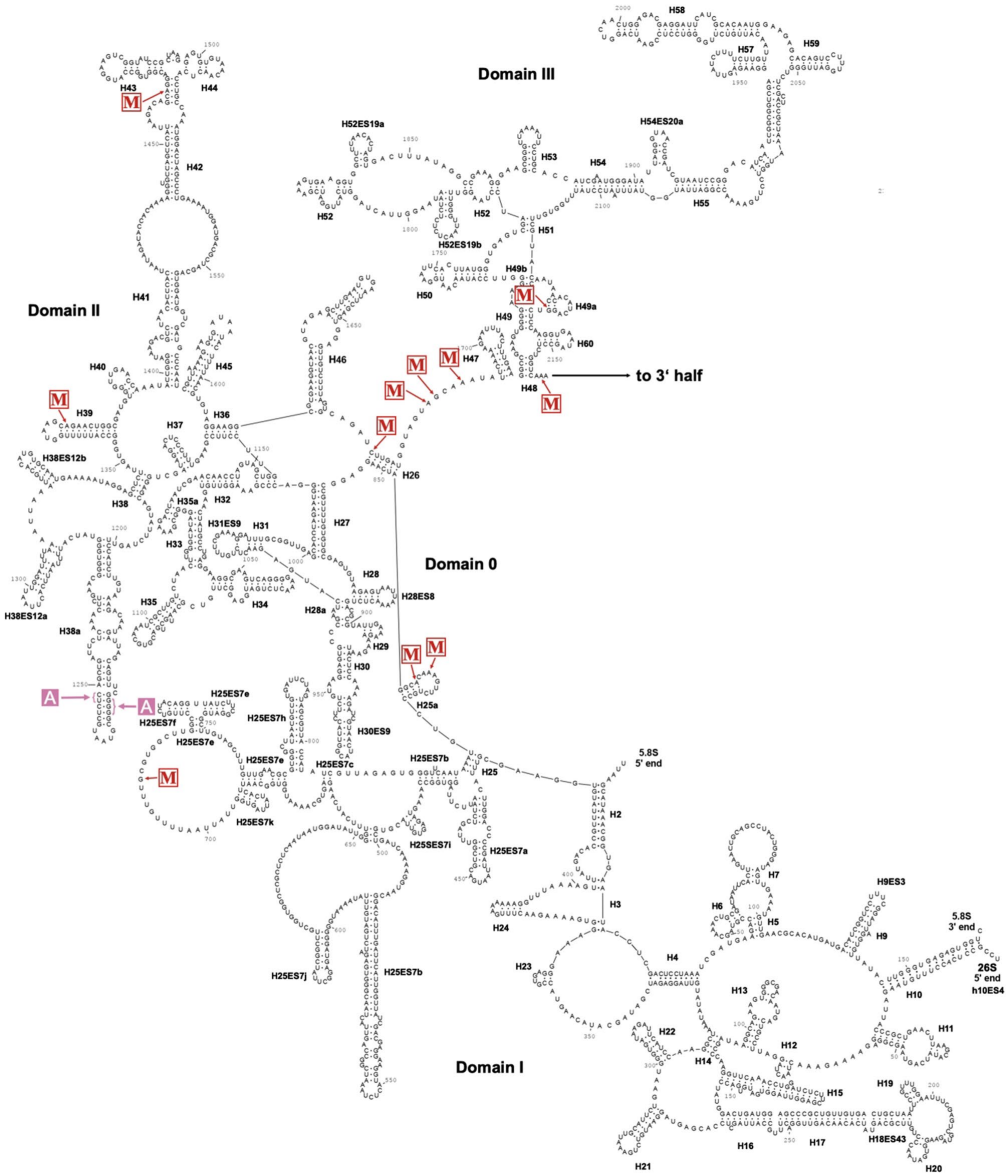

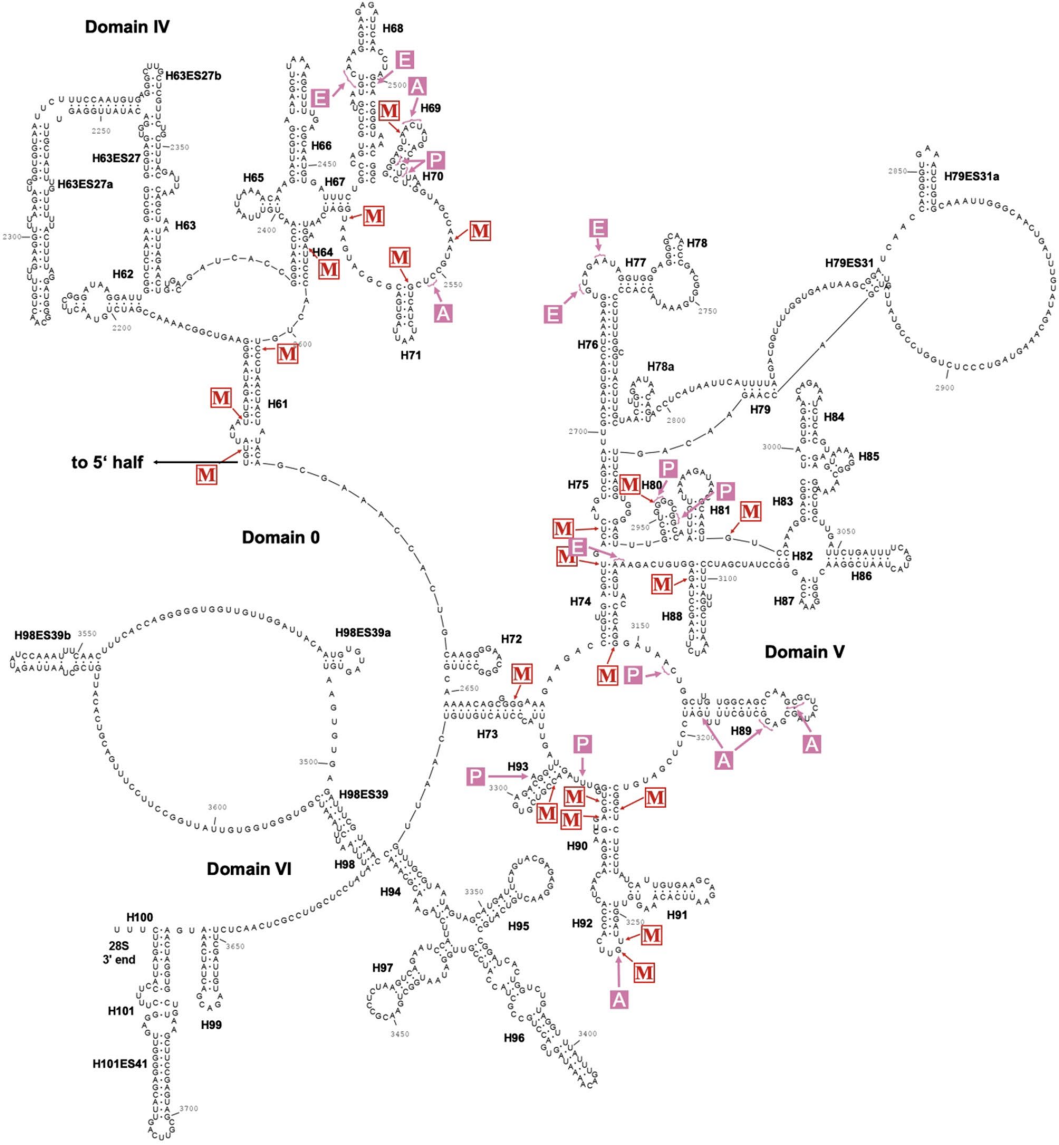
Secondary structure of the 26S rRNA of *D. discoideum* with 2′-*O*-Me sites. The secondary structure of the 26S rRNA was inferred by homology and drawn using RNAviz (v. 2.0.3). The 2′-*O*-methylated nucleotides as identified by RiboMeth-seq are marked with an arrow and ‘M’ (red). Nucleotides located in the A, P, and E sites of the ribosome are indicated in pink. Due to the size of the 26S rRNA, the figure is split into the 5′ half and 3′ half. The predicted interaction with the 5.8S rRNA is shown at the 5′ end. Helices (Hx) are named to convention and expansion segments (ESx) are labeled.

As methylated rRNA positions are required for folding and structural stabilization of rRNAs, thereby contributing to ribosome function^8^, it was of interest to localize the 2′-O-methylated positions in the context of the rRNA structure of *D. discoideum*. A partial structure of the large ribosomal subunit of *D. discoideum* has been published recently^71^, but no high-resolution structural data is available for complete ribosomes from any species of the Amoebozoa. To obtain a model for the rRNA secondary structures, we employed homology modelling using sequences of species from the evolutionary supergroups of Opisthokonta and Archaeplastida[31]. In brief, we aligned the rRNAs from the amoeba with the corresponding small and large subunits’ (SSU and LSU, respectively) rRNA sequences from A. thaliana, Caenorhabditis elegans, Drosophila melanogaster, and H. sapiens (Supplementary Table S3). The inferred secondary structure models of the 17S and 26S (with the 5.8S) rRNAs of *D. discoideum* are shown in Figs. 3 and 4, respectively, and include the 2′-O-methylated positions.

Central parts of ribosomes from different species are structurally highly conserved and variation appears restricted to peripheral regions and the so-called expansion segments (ES)^72^, which often harbor species-specific sequences. This is exactly what the models for the amoebal rRNA structures display (Figs. 3 and 4). This holds particularly true for the conserved regions involved in the formation of A, P and E sites. Not surprisingly, the ES of *D. discoideum*, which are not covered in the aforementioned structure^71^, exhibited significant differences as compared to the ES in other species (exemplified for *H. sapiens*; Supplementary Table S6).

About half of the 2′-O methylated positions were found in the vicinity of nucleotides residing in the A, P and E sites, and the other half in other regions of the rRNAs (Figs. 3 and 4). These latter positions localized frequently to formally single stranded regions, or to nucleotides at the very beginning of helical stems. When comparing the 2′-*O*-Me patterns in wild type *D. discoideum* to those in *S. cerevisiae*, *H. sapiens*, and *A. thaliana*, we found 28 of the 2′-*O*-Me sites conserved in at least one of these organisms, and therefore, the other 21 sites are specific to *D. discoideum* (Table 1). Only one of these positions, Gm711 in the 26S rRNA, was found in an ES (Fig. 4), indicating that 2′-*O*-Me is largely restricted to the core of the ribosome in *D. discoideum*. Noteworthy, five of the 13 specific 2′-*O*-Me sites on the 26S rRNA were locating in domain 0, which has been shown in other species to coordinate folding of all other domains of the LSU rRNA, including the peptidyl transferase center (PTC)^73^.

**Figure 5:**
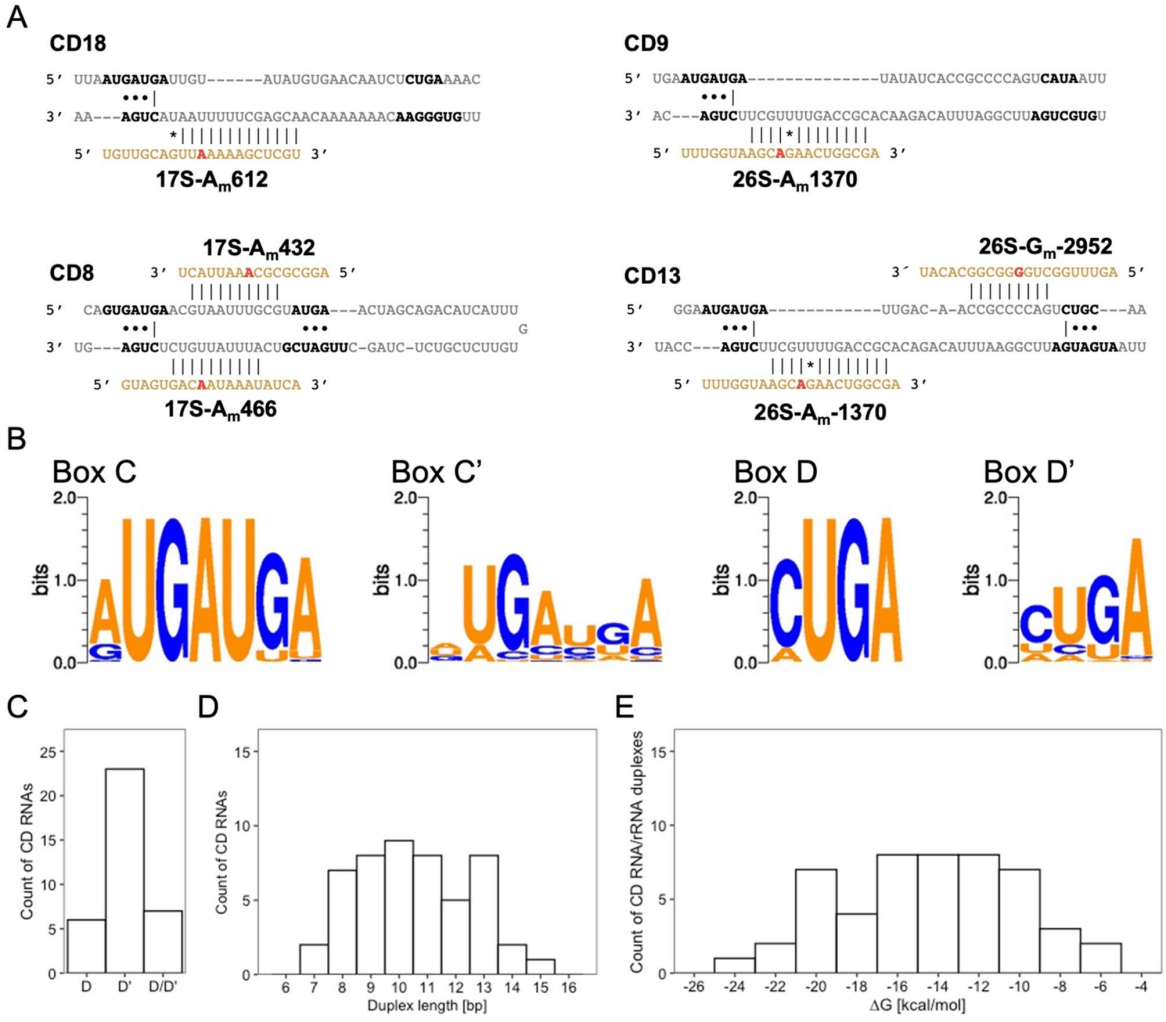
Features of CD RNAs in *D. discoideum*. (**A**) Examples of CD RNAs guiding 2′-*O*-Me at one or two rRNA positions. Single (top) and double (bottom) usage of D boxes of selected CD RNAs guiding positions in the 17S (left) and the 26S rRNA (right). Shown are CD RNA sequences (grey) with nucleotides involved in the formation of the k-turn (black). The guided part of the rRNA is shown in orange with the methylated residue highlighted in red. Intra- and intermolecular interactions are denoted for Watson–Crick (|) and G/U base pairing (*), as are the A/G and U/U base pairs (•) involved in the formation of the k-turn. (**B**) Conservation of C, C′, D and D′ box sequences shown with WebLogo^60^. (**C**) Distribution of CD RNAs using box D, D′ or both. Duplex lengths (in bp; **D**) and minimal free energies ΔG (in kcal/mol; **E**) of the interaction between CD RNA and the guided rRNA position.

### The majority of 2′-*O*-Me sites in *D. discoideum* can be associated to box C/D snoRNAs

To identify snoRNA guides for the 2′-O-methylated sites, we employed next RNAhybrid, since snoScan alone was not able to predict all targets for our set of box C/D snoRNAs (Fig. 2B). This resulted in the prediction of 46/49 2′-*O*-Me sites with at least one, occasionally two box C/D snoRNA guides (Table 1). The snoRNAs guiding 2′-O methylation at these rRNA sites were named CDx (x = natural numbers; Supplementary Table S5). For the remaining 9 box C/D snoRNAs, we could not assign a 2′-*O*-Me site in either rRNA, and therefore we classified these sequences as orphans, and named them accordingly ORx (Supplementary Table S5). Seven of the CD RNAs can make use of both their D and D′ boxes to guide 2′-*O*-Me in one or both rRNAs (Tables 1 and Supplementary Table S7). For most positions targeted by these CD RNAs, no alternative guides were found. Rather, CD1 and CD19 have two targets each for their D′ boxes, additional to the targets of their D boxes (Supplementary Table S7). The majority of CD RNAs, however, is predicted to employ either its D or D′ box. Figure 5A displays examples for single and double usage of D boxes, shown exemplarily for one case each in the 17S and 26S rRNA. The predicted bimolecular interactions of the CD RNAs with their rRNA targets are shown in Supplementary Figs. S2 and S3 for D and D′ box guides, respectively. Earlier work had shown the functionality of box C/D snoRNA in guiding 2′-O-Me in *D. discoideum* by primer extension at a low dNTP concentration^43^.

**Figure 6:**
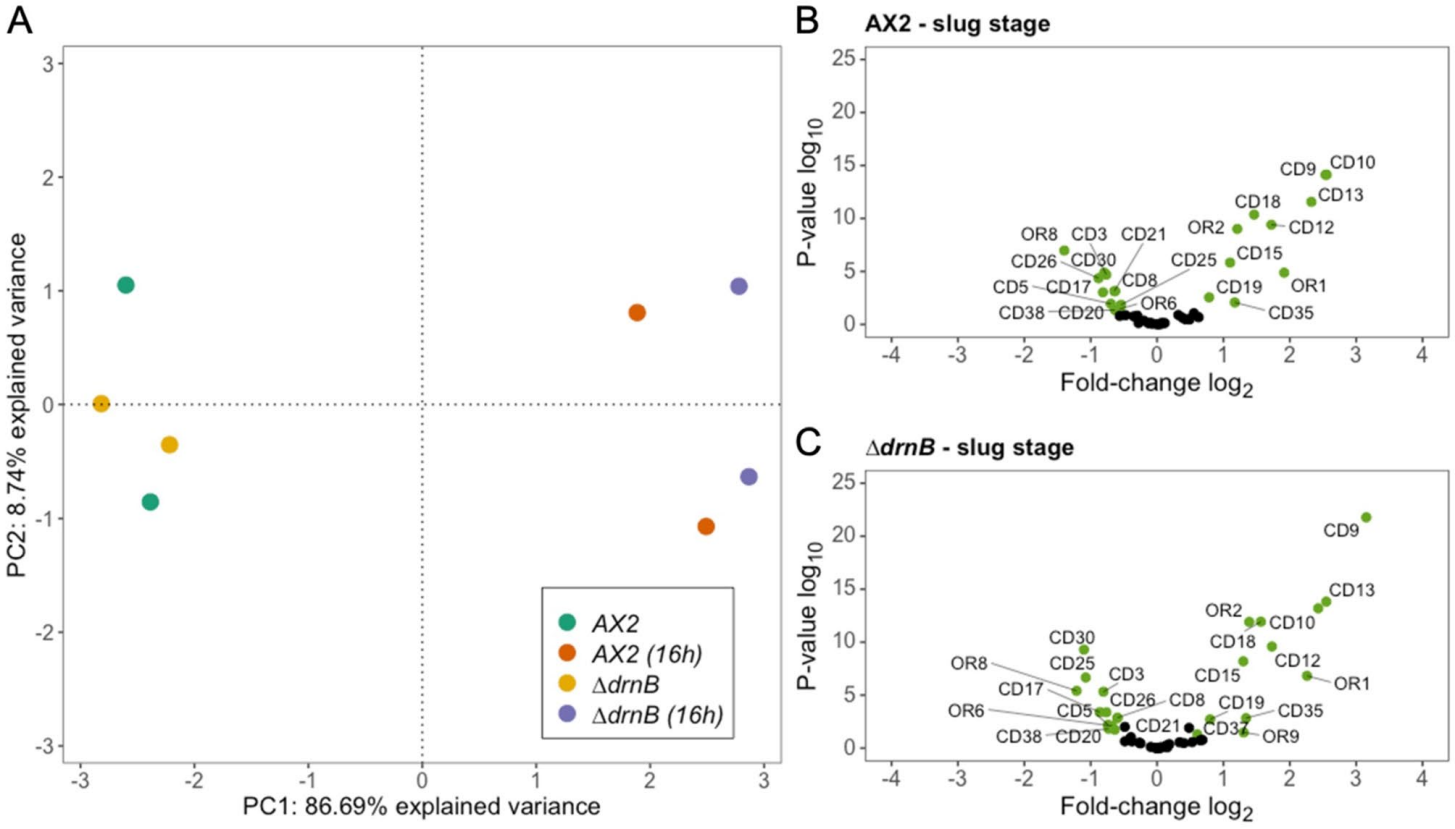
Analysis of box C/D snoRNA expression in axenic growth and development of the AX2 and *∆drnB* strains. (**A**) Principal component analysis (PCA) of data from RNA-seq on the indicated strains and conditions. Volcano plots of box C/D snoRNA expression changes in the slug stage of AX2 (**B**) and *∆drnB* (**C**). Significantly up- or downregulated box C/D snoRNAs are labelled and colored green.

### Features of box C/D snoRNAs and their interactions with rRNA

The box C/D snoRNAs in *Dictyostelium* are between 66 and 113 nt in length, with an average GC content of 32.2% and box C–D distances between 50 and 97 nt (Supplementary Table S5). The terminal stem often found in box C/D snoRNAs in other species (Fig. 1), is predicted by snoScan only in 25 of the 47 box C/D snoRNAs of *D. discoideum* (indicated with a positive TS score in Supplementary Table S4). In contrast, the box C and box D sequences forming the k-turn motif are highly conserved (Fig. 5B); in particular, the GA dinucleotides forming *trans* Hoogsteen/sugar-edge A•G base pairs are present in all CD RNAs selected by the described criteria (but not in all OR RNAs, see Supplementary Table S4). Furthermore, we found that almost all CD RNAs abide to the box D consensus sequence CUGA, with a small fraction of snoRNAs featuring an AUGA instead (Fig. 5B and Supplementary Table S4). Compared to these motifs, the box C′ and box D′ sequences show considerably more variation in *Dictyostelium* (Fig. 5B). Despite this, the majority of methylated positions is predicted to be guided by the D′ boxes of individual CD RNAs (Fig. 5C), similar to observations made for the human box C/D snoRNAs^12^. The lengths of the CD RNA/rRNA duplexes distributed around 11 bp within a range of 7–15 bp, with average minimal free energies (MFE) of − 13.9 kcal/mol (Fig. 5D,E). In these predicted CD RNA/rRNA interactions, we observe the frequent occurrence of G*U base pairs^74^, occasionally A/C base pairs^75^, and a single G/A mismatch (Supplementary Figs. S5 and S6). Only for the CD16/17S-G1589 duplex, we noticed that apparently the + 6 position is targeting, rather than the consensus + 5 position, as has also been observed before in other species^13^.

### Box C/D snoRNAs accumulate differentially during development of *D. discoideum*

Our primer extension experiments (Supplementary Fig. S2) indicated no 5′-end size heterogeneity of box C/D snoRNAs in *D. discoideum*. In absence of an internal control, a correlation between band intensity and expression levels is difficult. Furthermore, we could not obtain a product for several snoRNAs, despite the use of several distinct primers in these experiments. Therefore, to obtain a more complete view on box C/D snoRNA accumulation, we retrieved RNA-seq datasets for AX2 and *∆drnB* in axenic growth and in the slug stage of development from NCBI, which were originally deposited by Liao et al.^47^. As a first step, we performed a principal component analysis (PCA) of box C/D snoRNA expression on two biological replicates for each time point per strain. The analysis revealed global changes of box C/D snoRNA abundance in the development of the AX2 and *∆drnB* strains (Fig. 6A), however, not between AX2 and *∆drnB*. This is corroborated by comparative 2D plots of DESeq2-normalized reads of individual box C/D snoRNAs in the two strains and under the two growth conditions (Supplementary Fig. S7A). In a subsequent analysis of individual box C/D snoRNAs, we considered changes significant if an adjusted p-value < 0.05 and an at least 0.5-log_2_fold-change in RNA quantity was observed. Using these criteria, 22 box C/D snoRNAs were significantly up- or downregulated in the slug stage of development of AX2 (Fig. 6B,C and Supplementary Fig. S7B). In contrast to this and as seen before (Fig. 6A), we did not observe significant differences in the box C/D snoRNA between AX2 and *∆drnB* except for OR9 and CD37, which were upregulated in the slug stage in *∆drnB*, but not in AX2 (Fig. 6B,C and Supplementary Fig. S7B). For several box C/D snoRNAs we also performed Northern blot analyses (Supplementary Fig. S8) that confirmed at large the expression patterns seen by RNA-seq, in-line also with an earlier study employing Northern blotting on the 17 box C/D snoRNAs identified at the time^43^.

We wondered whether the changes that we observe in the 2′-*O*-Me patterns (Fig. 2) can be explained by differences in the accumulation of the guiding CD RNAs. This is clearly not the case, as a 2D plot of the DESeq2-normalized reads of CD RNAs against the RMS scores at all methylated sites revealed no correlation in axenic growth; rather, full and fractional methylation is observed independent of the CD RNAs’ abundance (Supplementary Fig. S7C). Furthermore, a 2D plot of the log_2_fold-change of the RMS score against the log_2_fold-change of CD RNA accumulation in the slug stage (Supplementary Fig. S7D) showed no differences. Thus, changes in the 2′-*O*-Me patterns can in general not be attributed to altered CD RNA amounts in the development of *D. discoideum*.

## Discussion

### Ribosome heterogeneity in Amoebozoa

In this study, we have investigated the 2′-*O*-Me landscape of *D. discoideum*’s rRNAs and associated box C/D snoRNAs. To our knowledge, this is the first comprehensive report on this topic for any species from the Amoebozoa, one of five eukaryotic evolutionary supergroups^31^. Using RMS^11^, we have identified 45 positions that are fully methylated in the rRNAs of the amoeba, and additionally 4 positions that exhibit a substoichiometric 2′-*O*-Me (Fig. 2 and Supplementary Fig. S4). This indicates that ribosome heterogeneity exists in Amoebozoa. Such variations in the chemical modification of nucleic acids making up the translation apparatus have been reported already for organisms from other evolutionary supergroups, in particular Opisthokonta^11–13,15,30^, but also in Archaeplastida^29^. With our data from a third evolutionary supergroup, the Amoebozoa, we suggest that ribosome heterogeneity represents a trait common to all eukaryotes.

Ribose methylation is thought to occur largely co-transcriptionally^11,76^. Thus, variation in the levels of this modification could be influenced by the rDNA organization. In *D. discoideum*, rRNAs are transcribed^42^ from extrachromosomal, palindromic elements^39,40^. Expression from extrachromosomal rDNA is rare, but described also, e.g., for *D. rerio*^14^. In the amoeba, clusters of the rDNA palindromes can condense into chromosome-like bodies^41^. This poses the question whether ribose methylation might be affected by limited accessibility for the snoRNPs to the nascent transcript. Our data indicates that the 2′-*O*-Me modification can be actually introduced equally well on rRNAs transcribed from extrachromosomal rDNA, as compared to chromosomally encoded transcripts.

A single 2′-O-methylated position, 26S-A1463, displayed altered RMS scores in the development of the amoeba and between the investigated strains (Fig. 2 and Supplementary Fig. S4). Such changes were also observed in the development of mouse^30^ and zebrafish^13^. Further, fractionally methylated sites in rRNA residues in cultured human cells became (close to) fully modified in differentiated tissues^70^. These aforementioned studies also all used RMS, as the preferred high-throughput analysis method of 2′-*O*-Me patterns, allowing for single nucleotide analysis in a quantitative manner, unlike alternative approaches. The advantages of RMS were also highlighted in a comparative study on rRNA from *Trypanosoma brucei* that further revealed 2′-*O*-Me patterns, which depended on the living conditions of the parasite^77^. Similar methodological advantages to RMS are also realized by the recently introduced and validated RiboMethSeq tool^78,79^ and the methylated positions reported here for the AX2 strain were at large confirmed independently using this method (Virginie Marchard and Yuri Motorin, personal communication).

For the majority of 2′-O-methylated rRNA positions, we have bioinformatically identified suitable CD RNAs (Fig. 1, Table 1). A subset of 17 such molecules had been reported earlier^43^, and we have added here additional 21 novel box C/D snoRNAs with a target in rRNAs, plus nine without. Previously, small non-coding RNAs in the amoeba were all called DdR-x (x = natural number), for *Dictyostelium discoideum* RNA^43^. With a functional association, we now have decided to rename the box C/D snoRNAs with an rRNA target to CDx (x = natural number), and those without to ORx RNA (for orphan).

### A secondary structure model for the ribosomal RNA in *D. discoideum*

For the localization of the 2′-O-methylated positions, we propose, additionally to the partial Cryo-EM structure of the nascent ribosome, here a complete model for the secondary structure of the large rRNAs in the amoeba (Figs. 3 and 4). This is based on a homology alignment of rRNA sequences from organisms of two evolutionary supergroups, the Opisthokonta and Archaeplastida^31^. In the rRNA models for the Amoebozoan *D. discoideum*, about half of the 2′-O-methylated nucleotides are found close to the A, P and E sites of the ribosome. The remainder localize either in formally single stranded regions or at the very beginning of helical stems where they presumably fulfil a stabilizing function or support rRNA folding. Our models of the *D. discoideum* rRNAs are greatly supported by the previously introduced Cryo-EM structure of the nascent 60S subunit of *Dictyostelium*^71^, that features parts of the proposed structural elements of the 26S rRNA (Fig. 4), while the ESs are not covered in this structure.

In *D. discoideum*, the 2′-O-methylated positions U3254 and G3255 on the 26S rRNA are orthologous to the methylated sites U2921 and G2922 in *S. cerevisiae* (Table 1). In yeast, G_m_2922 is highly important for the docking of transfer RNAs (tRNA) in the A-site via base pairing with C_75_ in their CCA-tail^80^. This suggests that G_m_3255 might fulfill the same function in *Dictyostelium*. U3254 is likely modified by the CD25 RNP (see also below), however, a guide for G_m_3255 is missing (Table 1). Intriguingly, position G2922 in *S. cerevisiae* is modified by the SAM-dependent methyltransferase Spb1, independent of a box C/D snoRNA guide^80^. *Dictyostelium*’s genome encodes the homologous *fsjC* gene (http://dictybase.org/gene/DDB_G0284945), and by analogy we hypothesize that its gene product might fulfil the same function as Spb1 in yeast. We can, however, not exclude that the CD25 RNP might also introduce that methylation by using its + 6 position, in analogy to two *D. rerio* snoRNPs that guide neighbouring positions in the rRNAs^13^.

### The box C/D snoRNA genes

Box C/D snoRNAs in *D. discoideum* are encoded in intergenic regions or as part of introns of protein-coding genes, and in either set-up, they can be generated from mono- or poly-cistronic transcriptional units^62^. The selected set of 38 CD RNAs and their encoding genes display overall features similar to those seen in the original 17 sequences^43^. We found all box C/D snoRNAs in intergenic regions except for CD38, which is encoded in an intron of *DDB_G0283293* (Supplementary Table S5).

Aspegren et al.^43^ had reported three bi-cistronic transcriptional units of snoRNAs being expressed in *D. discoideum*. We identified seven additional clusters with two or three box C/D snoRNA genes (Supplementary Fig. S3). One of the tri-cistronic clusters (on chromosome 5; Supplementary Fig. S3), had been reported to contain CD16 and CD5, but the central CD23 gene had not been noticed at the time^43^. A primary transcript of that cluster was not observed, but for the other three originally reported bi-cistrons, primary transcripts had been shown^43^. The former observation might be explicable if the CD16–CD23–CD5 tri-cistron consists of independent mono- or bicistronic transcription units. In summary, box C/D snoRNAs in *D. discoideum* appear predominantly encoded in intergenic regions, half each as mono- and poly-cistrons.

Not only in *D. discoideum*, but also in other species with three-digit intron sizes, like *A. thaliana*, *S. cerevisiae* or *Schizosaccharomyces pombe* are box C/D snoRNAs largely encoded by independent genes (Supplementary Table S8). By contrast, in eukaryotes with larger introns such as *D. melanogaster* or *H. sapiens*, snoRNAs are more frequently encoded in the intervening sequences of protein-coding genes^81^. Neither the global abundance of introns in protein-coding genes, nor their frequency/gene appear to be correlated with an “intronization” of the box C/D snoRNA genes (Supplementary Table S8). Instead, their number appears increased in the analyzed multicellular organisms compared to those that can exist as unicellular species. In the evolutionary tree, the Amoebozoa with *D. discoideum* branched off after the split of the Archaeplastida (*A. thaliana*) and before the separation of the Opisthokonta encompassing as diverse organisms as *D. melanogaster*, *H. sapiens*, *S. cerevisiae*, or *S. pombe*^31^. This current situation might be explained by snoRNA numbers and their intronization having evolved after the split of the individual supergroups to meet the needs of the individual organism.

### Interactions of CD RNAs with rRNAs in *D. discoideum*

A productive interaction between a box C/D snoRNA and its target has been suggested to require 7–20 base pairs, thereby allowing for G*U pairs and a few mismatches but excluding bulges^82^. However, only 10 base pairs actually fit in the substrate binding channel, as observed for an archaeal box C/D snoRNP^24^. Overall, the interactions that we are proposing for the CD RNA/rRNA pairs adhere to these rules (Supplementary Figs. S5 and S6). The minimum free energy for the formation of the duplexes (Fig. 5) is, however, considerably higher compared to *H. sapiens*^12^. At the same time, the lengths of the interactions do not differ as much. This discrepancy can be attributed to the frequent occurrence of G*U base pairs, the occasional presence of A/C base pairs, and a single G/A mismatch (see below) that are predicted in individual interaction pairs. G*U base pairs have been observed also in analogous pairs of other species^12,13^, and they can be isosteric to Watson–Crick base pairs^74^. However, their occurrence appears more frequent in the amoeba, and in the extreme case of the CD12/26S-U2580 interaction (Supplementary Fig. S6), 3/9 base pairs are G*U. In three predicted duplexes, we noted an A/C base pair that appeared to be confined to the 6th position upstream of the D box (CD7/26S-G711 and CD23/26S-C3292; Supplementary Fig. S5) or D′ box (CD28/17S-C1715; Supplementary Fig. S6). An A/C interaction can also substitute for a canonical Watson–Crick base pair, if the adenosine is protonated, i.e. A(+)/C^75^. Distinct from these is the single G/A mismatch seen in the CD29/17S-U1264 pair (Supplementary Fig. S5) that is likely to cause structural perturbations in the interaction, which possibly is counteracted by the overall 13 base pairs surrounding the mismatch. As had been observed before in zebrafish^13^, the methylated position 17S-G1589 appears to be guided by the + 6 position of CD16 (Supplementary Fig. S6). We noted that non-Watson–Crick interactions occur in all predicted pairs that result in a fractional, but also in some with complete methylation (Table 1 and Supplementary Figs. S5 and S6). However, the overall strength (or weakness) of the CD RNA/rRNA interaction in *D. discoideum* does not appear to correlate with the RMS score (Supplementary Fig. S9), similar to observations made in human cells^12^. The lower free energies observed for the resulting duplexes (Fig. 5E) might rather be explained by the lower optimal growth temperature of 21 °C of *D. discoideum*^32^, compared to yeast or humans. At this temperature, the inferred stabilities apparently warrant appropriate 2′-*O*-Me levels in the rRNAs in the amoeba (Fig. 2).

### Features of the box C/D snoRNAs

The mature box C/D snoRNAs in *D. discoideum* exhibit generally established characteristics of this class of ncRNAs (Fig. 1). A stable terminal stem, however, is absent in about half of the mature box C/D snoRNAs (Supplementary Table S4). Such stems are considered important for the recognition by the box C/D snoRNA processing machinery^21,22,26,64,83^. In *H. sapiens* or *Xenopus laevis*, a lack of the terminal stem in mature snoRNAs appears to be compensated by self-complementary sequences in their precursors^84,85^. This allows for productive interactions with the processing machinery, upon which these sequences are thought to be removed^21,22,26^. Also in *D. discoideum*, complementary stretches can be found up- and downstream of some box C/D snoRNAs without a terminal stem (data not shown). Therefore, we speculate that these sequences might be present in presumed precursor molecules.

*Dictyostelium discoideum* CD RNAs are predicted to use the antisense elements associated with the weakly conserved D′ box sequences more frequently than those with the highly conserved D boxes (Fig. 5B,C). The latter form, together with the in *D. discoideum* equally conserved C boxes, the terminal k-turn structure (Fig. 1), which is essential for maturation and assembly of the box C/D snoRNP complexes^86,87^. To some extent similar, a preferred usage of the D′ boxes in guiding 2′-*O*-Me to rRNA targets has also been reported for *H. sapiens* and *D. rerio*^12,13^. These studies revealed that in humans, the box C′ and D′ sequences displayed a considerably stronger conservation than seen for the amoeba, while in zebrafish box D′ was also less conserved and box C′ appeared degenerated.

**Figure 7:**
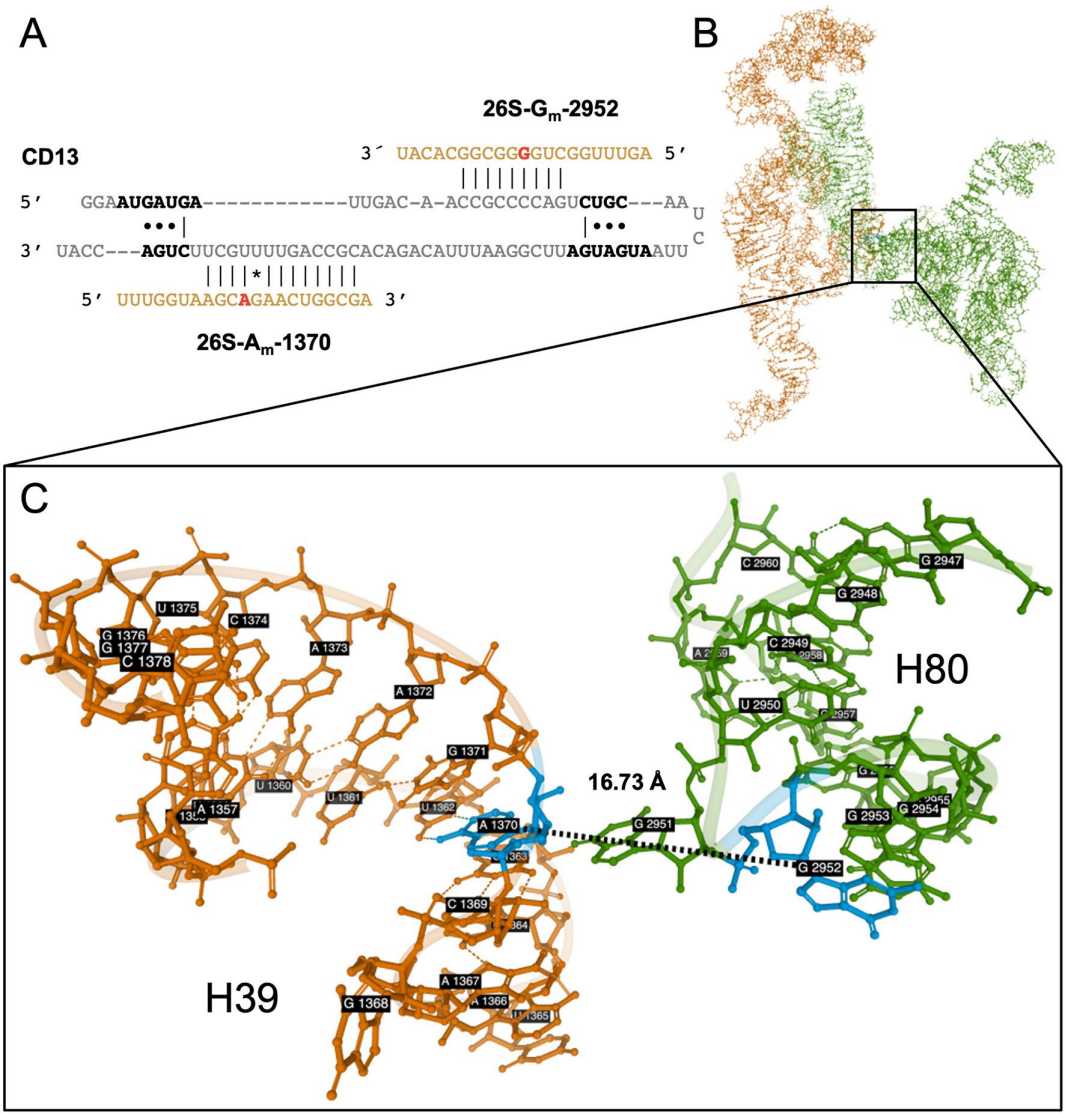
A model on the function of CD13 in guiding 2′-*O*-Me at two positions in the 26S rRNA. (**A**) Binary secondary structure of CD13 bound to positions A1370 and G2952 in the 26S rRNA of *D. discoideum*. (**B**) Scheme of relevant structure parts of the nascent 60S ribosomal subunit of *D. discoideum* (PDB accession: 5AN9) determined at 3.3 Å resolution via cryo-EM^71^. Domain II is displayed in orange and domain V in green (cf. Fig. 4). (**C**) Close vicinity (16.7 Å) of nucleotide A1370 in helix H39 and nucleotide G2952 in helix H80 (both positions colored in blue).

Seven CD RNAs of *D. discoideum* are predicted to utilize both antisense elements (Supplementary Table S7), with no paralogs or other box C/D snoRNAs known to be able to target the associated rRNA positions. At present, it is unknown, whether an interaction of both antisense elements with the target RNA(s) takes place simultaneously or sequentially. For *S. cerevisiae*, a simultaneous usage of both the antisense elements upstream the D and D′ boxes has been proposed, which might bring distant parts of the rRNA structure into proximity, thereby facilitating ribosomal maturation^88,89^. We wondered whether a similar situation might exist for “dual-use” CD RNAs in the amoeba. Since only a partial structure is available for the nascent 60S ribosomal subunit of *Dictyostelium*^71^, we inferred positions not included in that structure by homology to the human ribosome (PDB accession: 4UG0)^90^. Positions targeted by CD1, CD7 and CD19 (Supplementary Table S7) were not considered, as no orthologous methylated sites were found in other species (Table 1). CD25 of *D. discoideum* targets 17S-A612 and 26S-U3254 and the orthologous positions 18S-A668 and 28S-U4468 in the *H. sapiens* ribosome are around 100 Å apart, indicating sequential modification. Despite being distant in sequence, A1370 in helix H39 and G2952 in helix H80, which are both predicted targets of CD13 (Fig. 7A), lie only 16.7 Å apart in the available structure^71^ of the *D. discoideum* 60S subunit (Fig. 7B,C). That structure describes the large subunit at a late stage of maturation. It contains already helices H39 and H80, suggesting that the 2′-*O*-Me (not featured in the structure) must have taken place, as it requires the accessibility of the target sequences. We also cannot exclude that CD13 binds its targets after they reach proximity (Fig. 7). It is tempting to speculate, however, that the CD RNA actually might first spatially orient the target positions, then trigger their methylation, before the helices finally form. This would be supported by similar reports from *S. cerevisiae*^88,89^. Notably, in other species^2^, the orthologous nucleotides are part of the PTC, with G2952 being directly involved in the interaction with the CCA-tail of the tRNA residing in the ribosomal P site. The two predicted 26S rRNA targets of CD15 and CD19 (Supplementary Table S7) are so close that a simultaneous occupation of both positions would appear sterically challenging, if not impossible. On the other hand, it seems feasible that CD1 and CD8 might interact with their respective two predicted 17S positions (Supplementary Table S7) given their spacing. Thus, a simultaneous interaction with the two target sites appears unlikely for some of the “dual use” CD RNAs, but conceivable for others (CD1, CD8 or CD13).

### Alternative functions of *D. discoideum* box C/D snoRNAs?

We noted that a substantial set of 22 box C/D snoRNAs are differentially accumulated in the development of the amoeba compared to axenic growth, however, without manifesting in altered 2′-*O*-Me levels at the targeted positions (Fig. 6). This indicates that the amounts of CD RNAs are under either condition sufficient to warrant the appropriate 2′-*O*-Me levels (Supplementary Fig. S7C). Changes in the level of individual CD RNAs during development of the amoeba had already been observed in northern blots, e.g. for CD9, CD13 or CD15^43^. This is similar to data from *D. melanogaster*^91^ and *D. rerio*^13^. In the absence of an influence on 2′-*O*-Me levels in the amoeba (Supplementary Fig. S7D), developmental changes of many box C/D snoRNAs might instead point towards other physiological roles. Established is an alternative function as small Cajal Body RNAs (scaRNAs), which are structurally similar to box C/D snoRNAs, carrying an additional CAB box motif, but guide the sequence-specific methylation of small nuclear RNAs (reviewed for example in Refs.^16,92^). Also, some box C/D snoRNAs are involved in the processing of precursor rRNA molecules in a variety of organisms (summarized in Ref.^87^). While 2′-*O*-Me in tRNA is usually introduced by specialized stand-alone methyltransferases, e.g. Ref.^93^, certain positions are also guided by specific box C/D snoRNAs (reviewed in Ref.^16^), either alone or together with a dedicated box C/D scaRNA, like in the case of the wobble cytidine 34 of human tRNA^Met^^94^. Further functions that are conceivable also for *D. discoideum* box C/D snoRNAs encompass rRNA acetylation^88,95^, regulation of 3′ pre-mRNA processing^96,97^ or even the generation of small, sno-derived RNAs that might have regulatory functions, as described for other organisms^98–100^. Future work will show whether these possible functions are realized in *D. discoideum* by any of the OR RNAs or those CD RNAs, in which one antisense sequence lacks an identified rRNA target.

## Supplementary Information

Supplementary Information.

## Data Availability

The nucleotide sequence of the 17S rRNA of *D. discoideum *reported in this paper has been included in the GenBankTM/EBI Data Bank entry with accession number OK576654. The RMS data have been deposited under the GEO accession GSE186560.
