# Supplementary material for: Fractional 2′-O-methylation in the ribosomal RNA of Dictyostelium discoideum supports ribosome heterogeneity in Amoebozoa
Source: Sci Rep. 2022 Feb 4;12:1952. doi: 10.1038/s41598-022-05447-w (PMC8817022; doi:10.1038/s41598-022-05447-w)
Supplement: Supplementary file 1 — Supplementary Information. [file 41598_2022_5447_MOESM1_ESM.pdf]

Supplemental Data to

Fractional 2´-O-Methylation in the ribosomal RNA of *Dictyostelium discoideum* supports ribosome heterogeneity in Amoebozoa

Jan Diesend[1], Ulf Birkedal[2,3], Jonas Kjellin[4], Jingwen Zhang[1], Kim Philipp Jablonski[1], Fredrik Söderbom[4], Henrik Nielsen[2,5] and Christian Hammann[1,*]

[1] Ribogenetics Lab, Department of Life Sciences and Chemistry, Jacobs University gGmbH, Bremen, Germany
[2] Department of Cellular and Molecular Medicine, Copenhagen University, Copenhagen, Denmark
[3] present address: Department of Clinical Genetics, University Hospital Copenhagen, Denmark
[4] Department of Cell and Molecular Biology, Uppsala University, Box 596 Uppsala, S-75124 Sweden
[5] Genomics group, Nord University, Bodø, Norway

* corresponding author
Ribogenetics, Biochemistry Laboratory, Department of Life Sciences and Chemistry, Jacobs University Bremen gGmbH, Campus Ring 1, 28759 Bremen, Germany
c.hammann@jacobs-university.de

This file contains:

Table S1. RNA-seq data sets retrieved from SRA
Table S2. Oligonucleotides used in this study
Table S3. Accession numbers of rRNA sequences used in this study
Table S4. Parameters for snoScan and Classifier Scores
Table S5. Genomic location of C/D box snoRNAs with predicted rRNA methylation sites for CD RNAs
Table S6. Main differences of ribosomal expansion segments in *D. discoideum* compared to *H. sapiens*
Table S7. Targets of CD RNAs which utilize box D and box D´
Table S8. Intronization of box CD snoRNAs in selected eukaryotic organisms
Figure S1. Selection criteria for box C/D snoRNAs in *D. discoideum*.
Figure S2. Size estimation of box C/D snoRNAs in *Dictyostelium* using primer extension.
Figure S3. Novel genomic clusters of box C/D snoRNAs in *D. discoideum*.
Figure S4. Comparison of RMS scores in axenic growth and the development of AX2 and *ΔdrnB*.
Figure S5. Predicted base pairing between rRNA and CD RNAs utilizing the D box motif.
Figure S6. Predicted base pairing between rRNA and CD RNAs utilizing the D´ box motif.
Figure S7. Analysis of box C/D snoRNA expression in axenic growth and development of the AX2 and *ΔdrnB* strains, and the relationship to 2´-O methylation in rRNA.
Figure S8. Northern blot analysis of selected box C/D snoRNA in axenic growth and development of the AX2 and *ΔdrnB* strains.
Figure S9. 2D plot of RMS score against MFE.

Supplemental References

Table S1. RNA-seq data sets retrieved from SRA[a] [1]

Strain	Stage	Replicate	Accession
AX2	axenic	1	SRX3776204
AX2	axenic	2	SRX3776205
AX2	slug	1	SRX3776206
AX2	slug	2	SRX3776207
ΔdrnB	axenic	1	SRX3776208
ΔdrnB	axenic	2	SRX3776209
ΔdrnB	slug	1	SRX3776210
ΔdrnB	slug	2	SRX3776211

[a]https://www.ncbi.nlm.nih.gov/sra

Table S2. Oligonucleotides used in this study

#	Name	Sequence (5´ -> 3')
1	*CD1*	AACTTCTTAGTTTTGGTCAAC
2	*CD2*	AGTTTTTGGCTTAATTAAAAATTAAA
3	*CD3*	AGTTTTTGGCTTAATAAAATATTAAA
4	*CD4*	AGTAACTATGAATATAGAATCAC
5	*CD5*	AGGAAAATTTAGATAACGCAAA
6	*CD6*	GTTAAAGACCTTACCACAG
7	*CD7*	ATTTGTGCGAACACGGA
8	*CD8*	AGACAATAAATGATCGATCAAA
9	*CD9*	GTCAGAAGCAAAACTGG
10	*CD10*	TTAAATGGAAAATCGTTATAATCA
11	*CD11a/b*	AGACAATAAAAATAAGGAACAAA
12	*CD12*	TCAATTTTCGTCATAGGTTATA
13	*CD13*	AAGCAAAACTGGCGTGT
14	*CD14*	GGCTAAAGATCATCAACAG
15	*CD15*	AAGACTGTCGTGAGAAATC
16	*CD16*	AAATTTCCATTCAGCATGATT
17	*CD17*	TTGTAATTTTTAAGAATCGTCTAT
18	*CD18*	ACGTGTTAAAAAAGATGTCC
19	*CD19*	AGATCTTGATGTAAATTGGAAA
20	*CD20*	ATTCTATAAGTGTAAAATTAAATGAT
21	*CD21*	TGTTTTAAAAAAACAGCCAAAG
22	*CD22*	GAAAGAATAATGCATAGTCTC
23	*CD23*	AAGACTGTCGTGAGAAATC
24	*CD25*	TATTAAAAAGCTCGTTGTTTTTTT
25	*CD26*	ATGTTATAGAAGAAGTAATGTTTT
26	*CD27*	AGTCAAATGTATTATGTAGAATT
27	*CD28*	ACAAAAATTTTGTACACGTAATG
28	*CD29*	ATGGTGTTGCATGGTAAATA
29	*CD30*	TGCTTGACTACTAGATAGG
30	*CD31*	AGTTATTATAATTTGAATCAGCAA
31	*CD32*	GTCATCTATCATAAGTTTCAGC
32	*CD33*	TCGATTCGGTATCAATGAAGCT
33	*CD34*	ATTTCCGCATGACGATTCTCA
34	*CD35*	TCAACATGGCTTAATAGAATG
35	*CD36*	AGGGAGGCCGTTCTTATCAAT
36	*CD37*	GACAGTCATGACAAAGGTGCT
37	*OR6*	GATCGATCCCTCCCTGCCAGT
38	tRNA-Gln$_{UUC}$ NB probe	CTCCCCATTCGGGAATCGAA
39	CD6 NB	GGCCAACAATTTTCTCAGCAAGAC
40	CD9/13 NB	GGCGGTGATATATCATCATTCA
41	CD24 NB	ACAGAAACTCTAGTCTTTAGCTGT

Table S3. Accession numbers of rRNA sequences used in this study

Species	Subunit	Accession	length [nt]
A. thaliana	18S	X16077.1	1809
A. thaliana	25S	X52320.1	3539
C. elegans	18S	NR_000054.1	1754
C. elegans	26S	NR_000055	3509
D. discoideum	17S	FR733593.1	1871
D. discoideum	26S	FR733594.1	3741
D. melanogaster	18S	NR_133559.1	1995
D. melanogaster	28S	NR_133562.1	3970
H. sapiens	18S	NR_146146.1	1869
H. sapiens	28S	NR_003287.4	5070

Table S4. Parameters for snoScan and Classifier Scores

	Parameters and values from Scan								Parameters and values additionally used for Classifier Score (CS)			
RNA[a]	C box	C score	D box	D score	TS[b] length (bp)	TS[b] likely formed	TS[b] score	Scan Score[c]	RNAseq expression	C-D gap (nt)	Gap score	CS[d]
CD1	AUGAUGA	12.73	CUGA	8.05	6	Yes	0.27	21.05	15	50	5	41.05
CD2	AUGAUGA	12.73	CUGA	8.05	2	No	-1.65	19.13	15	68	5	39.13
CD3	AUGAUGA	12.73	CUGA	8.05	1	No	-1.90	18.88	15	68	5	38.88
CD4	GUGAUGA	10.76	CUGA	8.05	4	No	-0.93	17.88	15	90	5	37.88
CD5	AUGAUGA	12.73	CUGA	8.05	4	No	-0.93	19.85	15	65	5	39.85
CD6	AUGAUGA	12.73	CUGA	8.05	5	Yes	2.88	23.66	15	70	5	43.66
CD7	AUGAUGA	12.73	CUGA	8.05	6	Yes	2.61	23.39	15	81	5	43.39
CD8	GUGAUGA	10.76	CUGA	8.05	4	No	-0.91	17.90	15	73	5	37.90
CD9	AUGAUGA	12.73	CUGA	8.05	2	No	-0.95	19.83	15	65	5	39.83
CD10	AUGAUGA	12.73	CUGA	8.05	6	Yes	2.42	23.20	15	65	5	43.20
CD11b	AUGAUGU	7.48	CUGA	8.05	7	Yes	3.88	19.41	15	60	5	39.41
CD11a	AUGAUGU	7.48	CUGA	8.05	2	No	-1.65	13.88	15	60	5	33.88
CD12	GUGAUGA	10.76	CUGA	8.05	5	Yes	2.36	21.17	15	59	5	41.17
CD13	AUGAUGA	12.73	CUGA	8.05	3	Yes	0.52	21.30	15	66	5	41.30
CD14	AUGAUGA	12.73	CUGA	8.05	5	Yes	3.93	24.71	15	71	5	44.71
CD15	CUGAUGA	8.44	CUGA	8.05	1	No	-1.90	14.59	15	97	5	34.59
CD16	AUGAUGA	12.73	CUGA	8.05	3	Yes	0.14	20.92	15	62	5	40.92
CD17	AUGAUGA	12.73	CUGA	8.05	2	No	-0.62	20.16	15	54	5	40.16
CD18	AUGAUGA	12.73	CUGA	8.05	8	Yes	3.94	24.72	15	54	5	44.72
CD19	GUGAUGA	10.76	CUGA	8.05	5	No	-1.48	17.33	15	57	5	37.33
CD20	GUGAUGA	10.76	AUGA	3.77	1	No	-2.41	12.12	15	65	5	32.12
CD21	GUGAUGA	10.76	CUGA	8.05	5	Yes	0.77	19.58	15	59	5	39.58
CD22	GUGAUGA	10.76	CUGA	8.05	5	No	-1.56	17.25	15	61	5	37.25
CD23	AUGAUGA	12.73	CUGA	8.05	4	Yes	0.90	21.68	15	61	5	41.68
CD24	AUGAUGA	12.73	CUGA	8.05	4	No	-0.93	19.85	15	59	5	39.85
CD25	AUGAUGA	12.73	CUGA	8.05	7	Yes	1.98	22.76	15	62	5	42.76
CD26	AUGAUGA	12.73	CUGA	8.05	5	Yes	1.14	21.92	15	62	5	41.92
CD27	AUGAUGA	12.73	AUGA	8.05	6	Yes	0.18	20.96	15	63	5	40.96
CD28	AUGAUGU	7.48	CUGA	8.05	7	Yes	4.08	19.61	15	65	5	39.61
CD29	AUGAUUG	3.27	CUGA	8.05	7	Yes	1.78	13.10	15	66	5	33.10
CD30	AUGAUUA	7.81	AUGA	3.77	2	No	-0.62	10.96	15	74	5	30.96
CD31	AUGAUGA	12.73	CUGA	8.05	2	No	-0.62	20.16	15	61	5	40.16
CD32	AUGAUGA	12.73	CUGA	8.05	7	Yes	0.93	21.71	15	55	5	41.71
CD33	AUGAUUA	7.81	AUGA	3.77	2	No	-0.62	10.96	15	74	5	30.96
CD34	AUGAUUA	7.81	AUGA	3.77	3	No	-1.69	9.89	15	61	5	29.89
CD35	AUGAUGA	12.73	CUGA	8.05	4	No	-0.32	20.46	15	75	5	40.46
CD36	AUGAUGA	12.73	CUGA	8.05	4	Yes	0.08	20.86	15	74	5	40.86
CD37	AUGAUUA	7.81	AUGA	3.77	3	Yes	0.33	21.05	15	66	5	31.91
CD38	AUGAUGA	12.73	CUGA	8.05	4	Yes	0.81	17.55	15	74	5	41.59
OR1	AUGAUGA	12.73	CUGA	8.05	1	No	-1.90	21.09	15	71	5	38.88
OR2	GUGAUGA	10.76	CUGA	8.05	6	Yes	2.28	18.88	15	61	5	41.09
OR3	AUGAUGA	12.73	CUGA	8.05	1	No	-1.90	18.88	15	71	5	38.88
OR4	AUGAUGA	12.73	CUGA	8.05	1	No	-1.90	13.30	15	72	5	38.88
OR5	AUGCUGA	8.15	CUGA	8.05	2	No	-1.66	11.91	15	71	5	34.54
OR6	AUGAUUA	7.81	AUGA	3.77	6	Yes	1.72	21.59	15	70	5	33.30
OR7	AUGAAGA	7.52	CUGA	8.05	4	Yes	1.98	16.56	15	53	5	37.55
OR8	AUGAGGA	8.23	CUGA	8.05	5	Yes	0.28	15.30	15	60	5	36.56
OR9	CUGAUGA	8.44	CUGA	8.05	1	No	-1.19	14.54	15	83	5	35.30

[a] Sequences with predicted methylation sites in rRNA are named CDx. and those without ORx for ORphan (x: natural number)
[b] TS: terminal stem
[c] snoScan Score = C score + D score + TS score
[d] Classifier Score (CS) = snoScan Score + RNAseq expression + Gap score

Table S5. Genomic location of C/D box snoRNAs with predicted rRNA methylation sites for CD RNAs[a]

RNA	Chrom.[b]	Start nt	End nt	Strand	Length [nt]	GC%	Predicted methylation site(s)
CD1	3	4409414	4409479	+	66	36.36	26S-Gm2132; 17S-Um1456; 17S-Gm1506
CD2	5	2599916	2599999	-	84	34.52	26S-Gm2661
CD3	1	3858016	3858099	-	84	33.33	26S-Gm2661
CD4	3	1341617	1341723	+	107	32.71	26S-Am2522
CD5	5	2585362	2585443	-	82	31.71	26S-Gm2984
CD6	2	7681951	7682036	-	86	34.88	26S-Gm3148
CD7	5	2249625	2249721	-	97	44.33	26S-Am711; 17S-Cm991
CD8	4	4444847	4444936	-	90	38.89	17S-Am432; 17S-Am466
CD9	3	4409131	4409211	+	81	40.74	26S-Am1370; 26S-Am1463
CD10	2	5045624	5045704	+	81	24.69	17S-Am1133
CD11a	2	7539516	7539591	-	76	38.16	26S-Am3279
CD11b	2	7539236	7539311	-	76	36.84	26S-Am3279
CD12	5	1152274	1152354	-	81	28.40	26S-Am841; 26S-Um2580
CD13	5	2600248	2600331	-	84	44.05	26S-Am1370; 26S-Am1463; 26S-Gm2952
CD14	3	3885476	3885562	+	87	31.03	26S-Gm2554
CD15	4	59280	59392	+	113	36.28	26S-Cm3281
CD16	5	2586030	2586108	-	79	32.91	26S-Gm1686; 17S-1588
CD17	2	2854096	2854165	-	70	27.14	26S-Um2170
CD18	4	5364580	5364650	-	71	29.58	17S-Am28
CD19	4	1888032	1888103	+	73	32.88	26S-Cm1673; 26S-Cm1685; 17S-Am796
CD20	6	2022190	2022263	+	74	27.03	17S-Um1255
CD21	5	3090313	3090388	+	77	31.17	17S-Um571
CD22	5	3090012	3090090	+	79	32.91	26S-Um2687
CD23	5	2585693	2585770	-	78	33.33	26S-Cm3292
CD24	3	2159499	2159574	+	76	34.21	26S-Am844
CD25	2	5722386	5722463	-	78	28.21	26S-Um3254; 17S-Am612
CD26	4	5364890	5364967	-	78	28.21	26S-Cm2603
CD27	1	4758315	4758393	+	79	22.78	26S-Am2159
CD28	4	5364289	5364369	-	81	35.80	17S-Cm1715
CD29	5	3859231	3859312	+	82	31.71	17S-Um1264
CD30	3	2037101	2037191	-	91	35.16	26S-Um2683
CD31	3	5036106	5036180	-	75	18.67	26S-Um2164
CD32	2	3177912	3177988	+	71	28.17	26S-Um2170
CD33	5	479167	479234	+	68	29.41	26S-Am1689
CD34	5	4272206	4272283	-	78	21.79	26S-Am2592
CD35	5	2434788	2434879	+	92	32.61	17S-Cm38
CD36	2	5722103	5722190	-	91	32.97	26S-Gm3124
CD37	1	3858279	3858361	-	83	39.76	17S-Gm1266
CD38	4	474437	474527	+	91	25.27	26S-Cm3212
OR1	2	4524799	4524900	+	102	37.25	-
OR2	4	4445171	4445247	-	77	36.36	-
OR3	4	5385832	5385918	-	87	33.33	-
OR4	4	5384392	5384479	-	88	31.82	-
OR5	2	6880357	6880444	+	88	38.64	-
OR6	4	1682178	1682262	-	85	30.59	-
OR7	4	1496745	1496814	-	70	37.14	-
OR8	3	5321597	5321673	-	77	22.08	-
OR9	4	1663005	1663104	-	100	35.00	-

[a]All box C/D snoRNAs are encoded intergenically, except for CD38, which is encoded in an intron of DDB_G0283293. Sequences with predicted methylation sites in rRNA are named CDx, and those without ORx for ORphan (x: natural number).

[b]Chromsomal accessions at www.dictybase.org are DDB0232428 (chr. 1), DDB0232429 (chr. 2), DDB0232430 (chr. 3), DDB0232431 (chr. 4), DDB0232432 (chr. 5), and DDB0232433 (chr. 6).

Table S6. Main differences of ribosomal expansion segments in *D. discoideum* compared to *H. sapiens*

Subunit	Expansion segment	Difference
SSU	h9ES3	h9ES3a completely missing; h9ES3b conserved in structure; h9ES3c prolonged
	h21ES6	h21ES6a conserved. contains the *D. discoideum*-specific methylation site Am796; h21ES6b extended; h21ES6c missing; h21ES6d conserved
	h26ES	completely missing
	h39ES9	almost 70 nt longer
LSU	H9ES3	constituted by the loop of H9. 6 nt smaller
	H25ES7	H25ES7a: ca. 200 nt shorter; H25ES7b: ca. 160 nt shorter; H25ES7c: 6 bp shorter; H25ES7d: completely missing; H25ES7e: ca. 40 nt shorter; H25ES7f: ca. 70 nt shorter; H25ES7g: completely missing; H25ES7h: similar length. different structure; three additional helices (now called H25ES7i-k)
	H28ES8	stem loop 3 nt shorter
	H30ES9	similar length, different structure
	H31ES9	stem loop 4 nt longer
	H38ES10	completely absent
	H38ES12	H38ES12a corresponds to H38ES12 in human LSU. H38ES12a 2 bp shorter with additional bulge near helix terminus; Additional helix (now called H38ES12b).
	H45ES15	completely absent
	H52ES19	H52ES19a corresponds to H52ES19 in human LSU. H52ES19a is similar length. different structure; Additional helix (now called H52ES19b).
	H54ES20	H54ES20a 5 bp shorter in Dicty; H54ES20b completely missing
	H63ES27	Overall almost 570 nt shorter: H63ES27a is 280 nt shorter. H63ES27b is almost completely missing.
	H78ES30	completely absent.
	H79ES31	Size is similar, but less structured with H79ES31a being structurally conserved and H79ES31b not formed
	H98ES39	Overall 110 nt shorter and less structured with H98ES39a being 95 nt shorter and H98ES39b being 15 nt shorter

Table S7. Targets of CD RNAs which utilize box D and box D´

CD RNA	box D target	Domain	box D´ target	Domain	Distance [nt]
CD1	17S-U1456	3´ major	17S-G1506	3´ major	50
CD1	17S-U1456	3´ major	26S-A2132	III	-
CD7	26S-G711	I	17S-C991	C	-
CD8	17S-A466	5´	17S-A432	5´	34
CD13	26S-A1370	II	26S-G2952	V	1581
CD15	26S-C3292	V	26S-C3281	V	11
CD19	26S-C1673	II	26S-C1685	II	12
CD19	26S-C1673	II	17S-A796	C	-
CD25	17S-A612	5´	26S-U3254	V	-

Table S8. Intronization of box CD snoRNAs in selected eukaryotic organisms

Organism	Fraction of genes with introns	Average intron size	Introns per gene	Number of C/D snoRNA genes
D. discoideum	65.2% [a]	129 bp [a]	1.85 [a]	47 [b]
S. pombe	43.0% [2]	107 bp [3]	0.9 [4]	32 [5]
S. cerevisiae	5.0% [6]	256 bp [3]	0.05 [4]	46 [5]
D. melanogaster	80.0% [7]	1639 bp [8]	4.67 [9]	111 [5]
H. sapiens	97.0% [10]	3365 bp [11]	7.8 [12]	275 [5]
A. thaliana	79.1% [13]	168 bp [14]	4.8 [15]	185 [5]

[a] www.dictybase.org
[b] this study

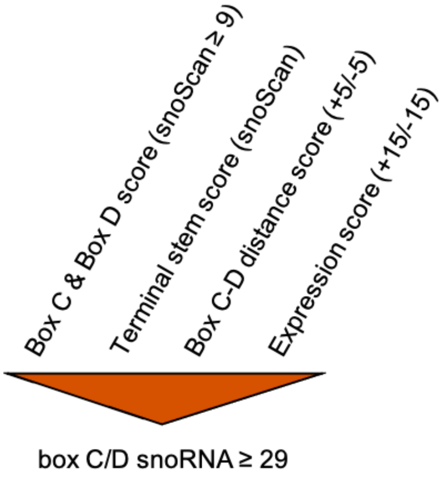

Figure S1. Selection criteria for box C/D snoRNAs in *D. discoideum*. Classification as box C/D snoRNAs based on snoScan scores for box C and box D, Box C-D distance and terminal stem score, augmented by score or penalty for RNA expression (Mapping to predicted loci and read coverage indicating distinct 5´ ends).

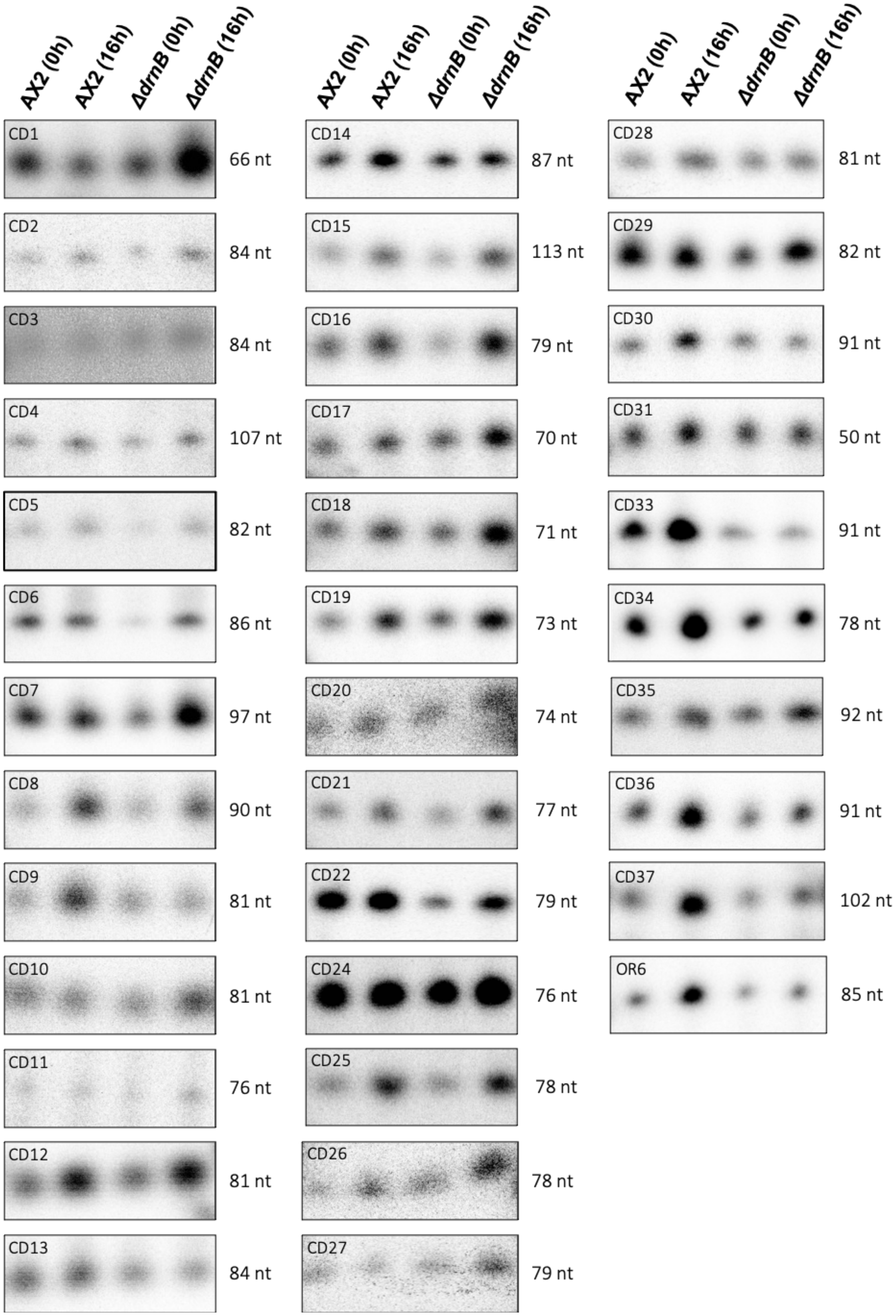

Figure S2. Size estimation of box C/D snoRNAs in *Dictyostelium* using primer extension. Shown are the primer extension products for the indicated box C/D snoRNAs in the Ax2 and *ΔdrnB* strains in axenic growth and in the slug stage of development. Inferred RNA sizes are shown to the right.

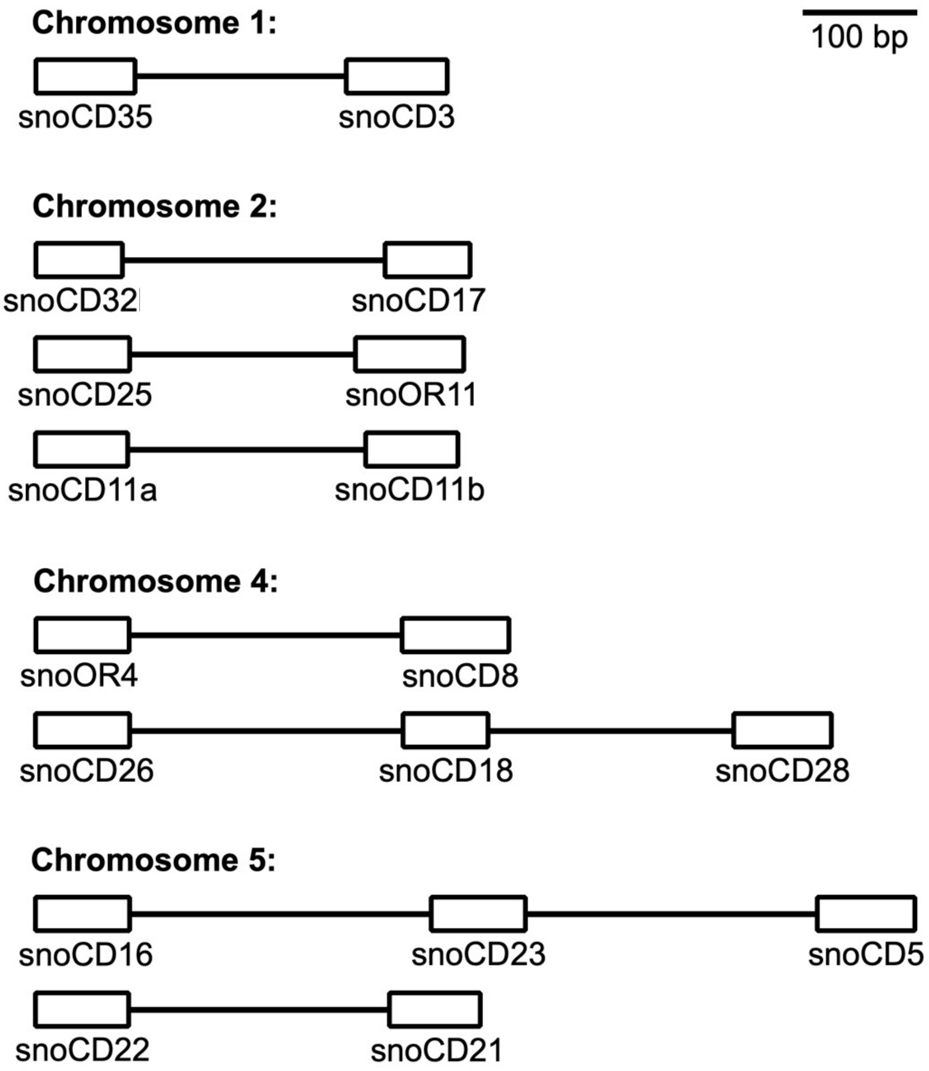

Figure S3. Novel genomic clusters of box C/D snoRNAs in *D. discoideum*. A scale is indicated on the upper right.

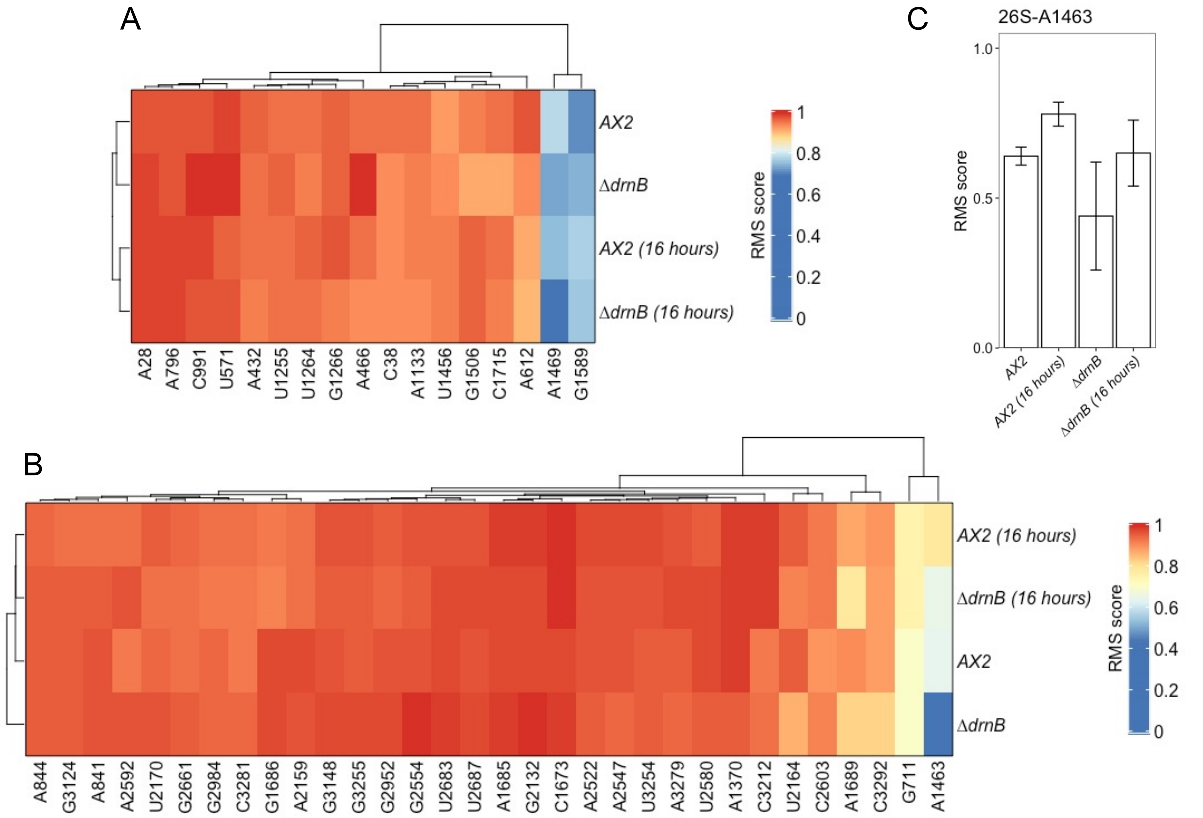

Figure S4. Comparison of RMS scores in axenic growth and the development of AX2 and *ΔdrnB*. Heatmap of RMS scores at all methylated positions on the 17S (A) and the 26S rRNA (B). (C) Example of a differentially methylated site between axenic growth and development of AX2 and *ΔdrnB*.

CD27 – 26S-A2159:

```
5’ UUGAUGAUGA-------------U … 3'
   |    •••|              |
3’ AC---AGUCUCAGUUUACAUAAUA … 5'
             |||||||||||||
   5’ GCCUCUGGUCAAAUGUAUUAA 3’
```

CD20 – 17S-U1255:

```
5’ UUUGUGAUGAAAU-----GUAUU … 3'
   |    •••|              |
3’ AC---AGUCUAAGAUAUUCAC-A … 5'
             |||||||||||
   5’ UUCAUGAUUCUAUAAGUGGUG 3‘
```

CD25 – 17S-A612:

```
5' UUAAUGAUGAUUGU------AUAUG … 3'
   ||    •••|
3' AA---AGUCAUAAUUUUUCGAGCAA … 5'
             *|||||||||||||
   5' UGUUGCAGUUAAAAAGCUCGU 3'
```

CD8 – 17S-A466:

```
5'  CAGUGAUGAACGUAAUUUGC … 3'
    |   •••|
3' UG---AGUCUCUGUUAUUUAC … 5'
             ||||||||||
     5' GUAGUGACAAUAAAUAUCA 3'
```

CD19 – 26S-C1673:

```
5’ UUGGUGAUGAAAAAAAUUUGCUACU … 3‘
   |    •••|              |
3’ AC---AGUCUCUAGAACUACAUUUA … 5‘
             ||||||||||
   5’ UAGUGCAGAUCUUGAUGGUAG 3‘
```

CD4 – 26S-A2522:

```
5’  UAAGUGAUGAUAACCCAAUAGA … 3'
     |    •••|
3’ UUU---AGUCUCAUUGAUACU- … 5'
              ||||||||||
    5’ GGCGGGAGUAACUAUGACUCU 3’
```

CD9 – 26S-A1370:

```
5’ UGAAUGAUGA-------------- … 3'
   ||    •••|
3’ AC---AGUCUUCGUUUUGACCGCA … 5'
             ||||*||||||||
   5’ UUUGGUAAGCAGAACUGGCGA 3’
```

CD13 – 26S-A1370:

```
5’    GGAAUGAUGA------------UU … 3'
      ||    •••|            |
3’ UACC---AGUCUUCGUUUUGACCGCA … 5'
               ||||*||||||||
     5’ UUUGGUAAGCAGAACUGGCGA 3’
```

CD1 – 17S-U1456:

```
5’ UGAAUGAUGAAACAAACAGA … 3'
    |    •••|
3’ UC---AGUCUUGAAGAAUCA … 5'
             |||||||||
   5’ AAUUAAAACUUCUUAGAGGGA 3’
```

CD30 – 26S-U2683:

```
5’ AAAAUGAUUAAAACGAUUUUGAUC … 3'
   ||    •••|
3’ UU---AGUAACGAACUGAUGAUCU … 5'
             ||||||||
   5’ CUGUUGAGCUUGACUCUAGUC 3’
```

CD23 – 26S-C3292:

```
5' A-AUAUGAUGAA-G-UAAUU-----C … 3'
   |       •••|
3’ UAC---AGUCUUCUGACAGCACUCU- … 5'
              |||| ||||||||||
    5' UGGGUUUAGACCGUCGUGAGA 3'
```

CD9 – 26S-A1463:

```
5' UGAAUGAUGA-------------- … 3'
   ||    •••|
3’ AC---AGUCUUCGUUUUGACCGCA … 5'
             ||||**||
   5’ UUAAGACAGCAGGACGGUGGC 3’
```

Figure S5. Predicted base pairing between rRNA and CD RNAs utilizing the D box motif. Shown are part of the rRNA (beige) with the methylated residue (red), and base pairs with relevant sequences of the guiding snoRNAs (grey) and their predicted C and D boxes (bold). Watson-Crick (|) and wobble base pairing (*) between rRNA and snoRNA are indicated. The predicted k-turns formed via non-Watson-Crick base pairing between C/D boxes are designated by (•). Except for the CD29 - 17S-U1264 pair, all k-turns follow the consensus. Pairs are sorted by decreasing stability of the duplex.

CD13 – 26S-A1463:

```
5’   GGAAUGAUGA------------UU … 3'
     ||   •••|              |
3’ UACC---AGUCUUCGUUUUGACCGCA … 5'
               ||||**||
     5’ UUAAGACAGCAGGACGGUGGC 3’
```

CD7 – 26S-G711:

```
5' UGAAUGAUGAUUGG-----------U … 3'
    |   •••|
3' CC---AGUCUAAACACGCUUGUGCCU … 5'
             |||| |*|*
     5' UUUUGUUUGCGUGGCUUGGCC 3'
```

CD29 – 17S-U1264:

```
5‘ CAAAUGAUUGAAAACCAAUA-UUUAUA … 3'
    |   •••
3’ CU---AGUCUACCACAACGUACCAUUU … 5'
             ||||| ||||||||
     5’ AUAAGUGGUGGUGCAUGGUC 3’
```

Figure S5. Predicted base pairing between rRNA and CD RNAs utilizing the D box motif; ctd.

CD35 – 17S-C38:

```
3' GAAUUAGAAACUCUG 5'
     |||||||||||||
5' UUUAAUCUUUGAGAACUAA 3'
```

CD7 – 17S-C991:

```
3' CAUAGACUAGCAGAA 5'
     |||||||||||||
5' UUAUCUGAUCGUCUGUUGA 3'
```

CD31 – 26S-U2164:

```
3' GAUGUAAUUAUGUAA 5'
     |||||||||||||
5' AUACAUUAAUACAUUCUUA 3'
```

CD32 – 26S-U2170:

```
3' GAAUAGAUGUAAUU 5'
     ||||||||||||
5' AUUAUCUACAUUAGCUGA 3'
```

CD10 – 17S-A1133:

```
3' GGCAGUUAAGGAAAUUC 5'
    *||||||||||||||
5' AUGUCAAUUCCUUUAACAUGA 3'
```

CD18 – 17S-A28:

```
3' CUGUUCGUAUACU 5'
     |||||||||||
5' UACAAGCAUAUGUCUGA 3'
```

CD17 – 26S-U2170:

```
3' AAUAGAUGUAAUU 5'
     |||||||||||
5' AUAUCUACAUUAGCUGA 3'
```

CD14 – 26S-G2554:

```
3' AAUCUACUGCUCC 5'
     |||||||||||
5' CUAGAUGACGAGUCAUA 3'
```

CD38 – 26S-C3213:

```
3' AUUCUUCUCGGCU 5'
     |||||||||||
5' AAAGAAGAGCCGUAUUA 3'
```

CD36 – 26S-G3124:

```
3' AAGACUGUGGAGAUCC 5'
     ||||||||*|||||
5' UUCUGACACUUCUAGAGUGA 3'
```

CD8 – 17S-A432:

```
3' UCAUUAAACGCG 5'
     ||||||||||
5' CGUAAUUUGCGUAUGA 3'
```

CD19 – 26S-A1685:

```
3' AUAAACGAUGAU 5'
     ||||||||||
5' AAUUUGCUACUGCUGC 3'
```

CD34 – 26S-G2592:

```
3' CACCCUUAGAGU 5'
     ||||||||||
5' AUGGGAAUCUCUCUUA 3'
```

CD26 – 26S-C2603:

```
3' UCAAUCCCUGUC 5'
     ||||||||||
5' UGUUAGGGACAUCUUA 3'
```

CD1 – 17S-G1506:

```
3' GUGUCUGGACAA 5'
     |||||||||
5' AACAGACCUGAUCUGA 3'
```

CD33 – 26S-A1689:

```
3' CUUAUAACGA 5'
     |||||||||
5' CAAUAUUUGCCCUGA 3'
```

Figure S6. Predicted base pairing between rRNA and CD RNAs utilizing the D´ box motif. Shown are part of the rRNA (beige) with the methylated residue (red), and base pairs with relevant sequences of the guiding snoRNAs (grey) and their predicted D box (bold). Watson-Crick (|) and wobble base pairing (*) between rRNA and snoRNA are indicated. As the k-loop is less well conserved, the sequences of the C´ boxes are not shown. Pairs are sorted by decreasing stability of the duplex. Position 17S-G1589 is exceptional being targeted by the +6 position of CD16 (marked).

```
CD13 - 26S-G2952:

3'  CGGCGGGGUCG  5'
     |||||||||
5'  ACCGCCCCAGUCUGC  3'
```

```
CD6 - 26S-G3148:

3'  CAAUAGGGACA  5'
     |||||||||
5'  UUUAUCCCUGUCUUA  3'
```

```
CD24 - 26S-A844:

3'  GAACUAGGCACAAA  5'
     |||||*||||||
5'  UUUGAUUCGUGUUUCUGA  3'
```

```
CD2 - 26S-G2661:

3'  AGAAGAAAGGGGCG  5'
     *|||||||||||
5'  AUUUCUUUCCCCGUCCGA  3'
```

```
CD3 - 26S-G2661:

3'  AGAAGAAAGGGGCG  5'
     *|||||||||||
5'  AUUUCUUUCCCCGUCCGA  3'
```

```
CD16 (+6) - 17S-G1589:

3'  UUACUAAUGGGUUA  5'
     *|||||||*|||
5'  UGUGAUUACUCAAUUUGA  3'
```

```
CD21 - 17S-U571:

3'  CUUAAUGGCG  5'
     ||||||||
5'  UAAUUACCGUCCGA  3'
```

```
CD37 - 17S-G1266:

3'  UACGUGGUGG  5'
     ||||||||
5'  UUGCACCACUCUGC  3'
```

```
CD1 - 26S-G2132:

3'  CUCUGGACUA  5'
     ||||||||
5'  CAGACCUGAUCUGA  3'
```

```
CD16 - 26S-G1686:

3'  UUAUAAACGAUGA  5'
     ||||||||||*
5'  UAUAUUUGCUAUUCUUA  3'
```

```
CD22 - 26S-U2687:

3'  GUCUGAUCUCAGU  5'
     ||||||||||*
5'  AAGACUAGAGUUUCUGU  3'
```

```
CD15 - 26S-C3281:

3'  AUUUGGGUCGAGU  5'
     ||||*||||||
5'  CAAACUCAGCUCUAUGA  3'
```

```
CD19 - 17S-A796:

3'  UACGACGUU  5'
     |||||||
5'  CUGCUGCAUUUAA  3'
```

```
CD5 - 26S-G2984:

3'  ACCUGUGAA  5'
     |||||||
5'  CGGACACUUUUGA  3'
```

```
CD25 - 26S-U3254:

3'  CCACUUGUUAGG  5'
     ||||||||||
5'  UGUGAACAAUCUCUGA  3'
```

```
CD11 - 26S-A3279:

3'  UGGGUCGAGUGC  5'
     |*||||||||
5'  CCUCAGCUCACACCGA  3'
```

Figure S6. Predicted base pairing between rRNA and CD RNAs utilizing the D´ box motif; ctd.

CD28 – 17S-C1715:<br><br>3′ CGCUGCC**C**GCCA 5′<br>*|||| |||| 5' UUGACGAGCGGA**CUGA** 3'	CD12 – 26S-A841:<br><br>3′ GGCACA**A**AGUU 5′<br>*|||||||| 5' AUGUGUUUCAG**AUGA** 3'
CD12 – 26S-U2580:<br><br>3' ACUAGG**U**AAGU 5' |||**|||*<br>5' AGAUUUAUUUC**AUGU** 3'	

Figure S6. Predicted base pairing between rRNA and CD RNAs utilizing the D´ box motif; ctd.

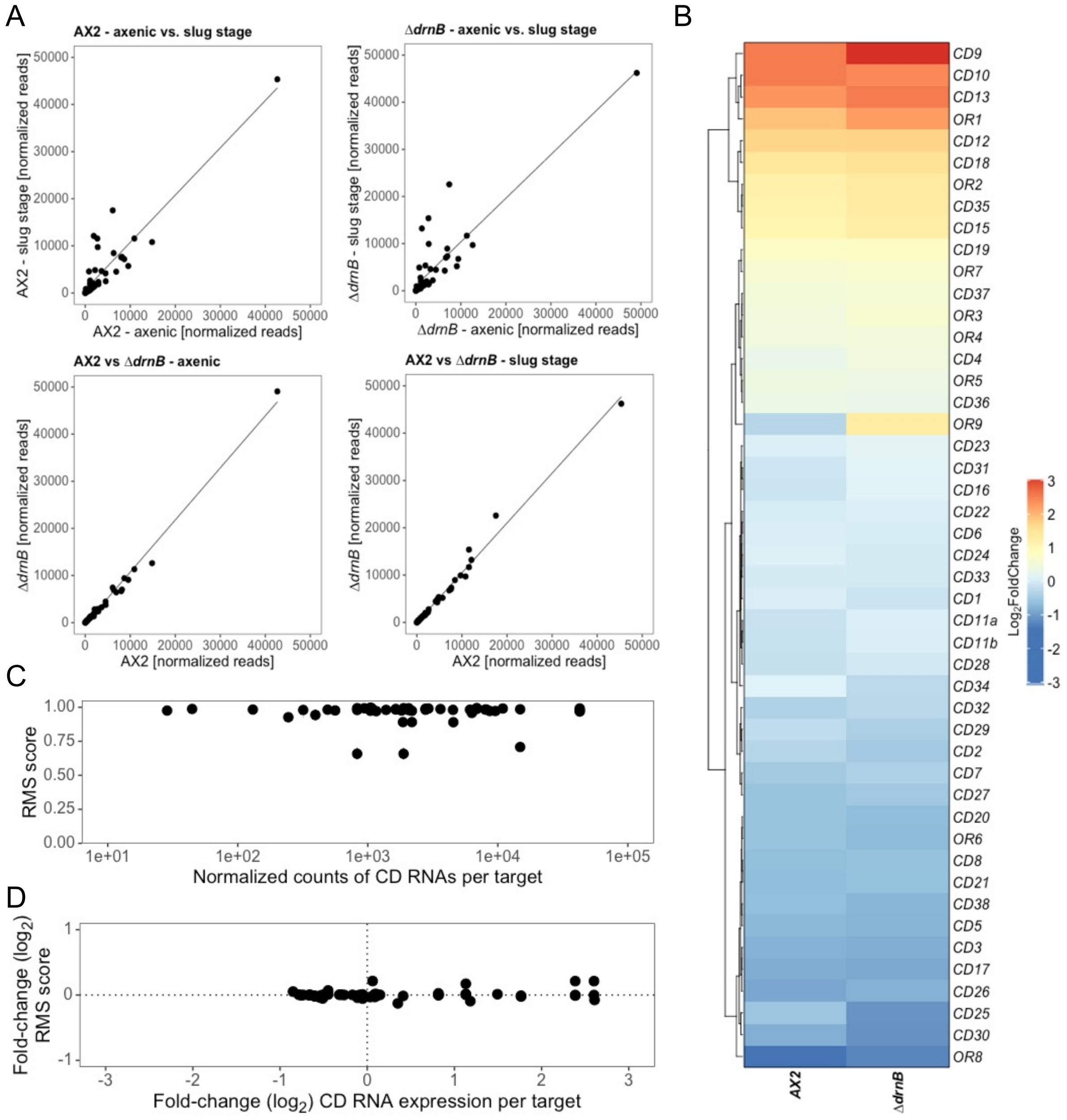

Figure S7. Analysis of box C/D snoRNA expression in axenic growth and development of the AX2 and *ΔdrnB* strains, and the relationship to 2´-O methylation in rRNA. (A) 2D plots of DESeq2-normalized reads in AX2 and *ΔdrnB* in axenic growth vs. slug stage (top) and in axenic growth or the slug stage of development of AX2 vs. *ΔdrnB* (bottom). (B) Heatmap of log_2 fold-change of box C/D snoRNA expression in the slug stage of development in the indicated strains. (C) 2D plot of log_2 fold-change of box C/D snoRNA expression per target in development and log_2 fold-change of the RMS score at all predicted methylation sites. (D) 2D plot of DESeq2-normalized counts of box C/D snoRNAs and RMS score at all predicted methylated sites.

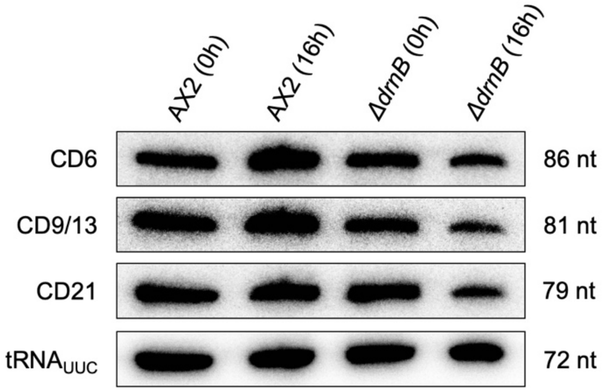

Figure S8. Northern blot analysis of selected box C/D snoRNA in axenic growth and development of the AX2 and *ΔdrnB* strains. The experiment was performed for snoCD6, snoCD9/13, and snoCD21 on RNA isolated from AX2 and ΔdrnB cells in axenic growth and after 16 hours of development. Due to their high sequence similarity, snoCD9 and snoCD13 could not be detected separately. Strains and developmental status are indicated above and the sizes of the tested CD RNAs are shown on the right. The $tRNA_{UUC}$ was used as loading control.

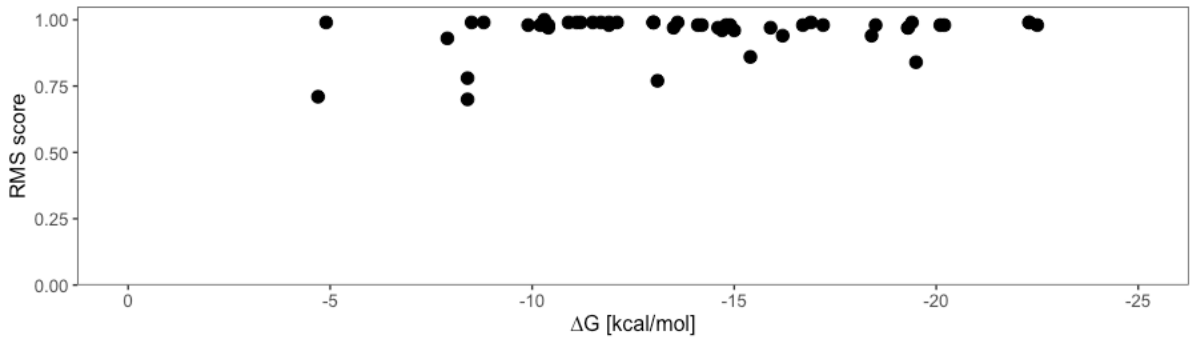

Figure S9. 2D plot of RMS score against MFE. Shown is the minimal free energy (ΔG in kcal/mol) and the RMS score of each CD RNA/rRNA interaction in *D. discoideum.*

Supplemental References

1 Liao, Z., Kjellin, J., Hoeppner, M. P., Grabherr, M. & Soderbom, F. Global characterization of the Dicer-like protein DrnB roles in miRNA biogenesis in the social amoeba Dictyostelium discoideum. *RNA Biol* **15**, 937-954, doi:10.1080/15476286.2018.1481697 (2018).

2 Wood, V. *et al.* The genome sequence of Schizosaccharomyces pombe. *Nature* **415**, 871-880, doi:10.1038/nature724 (2002).

3 Kupfer, D. M. *et al.* Introns and splicing elements of five diverse fungi. *Eukaryot Cell* **3**, 1088-1100, doi:10.1128/EC.3.5.1088-1100.2004 (2004).

4 Fair, B. J. & Pleiss, J. A. The power of fission: yeast as a tool for understanding complex splicing. *Curr Genet* **63**, 375-380, doi:10.1007/s00294-016-0647-6 (2017).

5 Dieci, G., Preti, M. & Montanini, B. Eukaryotic snoRNAs: a paradigm for gene expression flexibility. *Genomics* **94**, 83-88, doi:10.1016/j.ygeno.2009.05.002 (2009).

6 Neuveglise, C., Marck, C. & Gaillardin, C. The intronome of budding yeasts. *C R Biol* **334**, 662-670, doi:10.1016/j.crvi.2011.05.015 (2011).

7 Swinburne, I. A. & Silver, P. A. Intron delays and transcriptional timing during development. *Dev Cell* **14**, 324-330, doi:10.1016/j.devcel.2008.02.002 (2008).

8 ftp://ftp.flybase.net/genomes/Drosophila_melanogaster/current/fasta/dmel-all-intron-r6.16.fasta.gz.

9 Malko, D. B., Makeev, V. J., Mironov, A. A. & Gelfand, M. S. Evolution of exon-intron structure and alternative splicing in fruit flies and malarial mosquito genomes. *Genome Res* **16**, 505-509, doi:10.1101/gr.4236606 (2006).

10 Grzybowska, E. A. Human intronless genes: functional groups, associated diseases, evolution, and mRNA processing in absence of splicing. *Biochem Biophys Res Commun* **424**, 1-6, doi:10.1016/j.bbrc.2012.06.092 (2012).

11 Lander, E. S. *et al.* Initial sequencing and analysis of the human genome. *Nature* **409**, 860-921, doi:10.1038/35057062 (2001).

12 Sakharkar, M. K., Chow, V. T. & Kangueane, P. Distributions of exons and introns in the human genome. *In Silico Biol* **4**, 387-393 (2004).

13 Yan, H., Dai, X., Feng, K., Ma, Q. & Yin, T. IGDD: a database of intronless genes in dicots. *BMC Bioinformatics* **17**, 289, doi:10.1186/s12859-016-1148-9 (2016).

14 Arabidopsis Genome Initiative. Analysis of the genome sequence of the flowering plant Arabidopsis thaliana. *Nature* **408**, 796-815, doi:10.1038/35048692 (2000).

15 Atambayeva, S. A., Khailenko, V. A. & Ivashchenko, A. T. Intron and exon length variation in Arabidopsis, rice, nematode, and human. *Molecular Biology* **42**, 312, doi:10.1134/S0026893308020180 (2008).
